# Nexin-Dynein regulatory complex component DRC7 but not FBXL13 is required for sperm flagellum formation and male fertility in mice

**DOI:** 10.1371/journal.pgen.1008585

**Published:** 2020-01-21

**Authors:** Akane Morohoshi, Haruhiko Miyata, Keisuke Shimada, Kaori Nozawa, Takafumi Matsumura, Ryuji Yanase, Kogiku Shiba, Kazuo Inaba, Masahito Ikawa

**Affiliations:** 1 Research Institute for Microbial Diseases, Osaka University, Osaka, Japan; 2 Graduate School of Medicine, Osaka University, Osaka, Japan; 3 Graduate School of Pharmaceutical Sciences, Osaka University, Osaka, Japan; 4 Shimoda Marine Research Center, University of Tsukuba, Shizuoka, Japan; 5 The Institute of Medical Science, The University of Tokyo, Tokyo, Japan; Washington University School of Medicine, UNITED STATES

## Abstract

Flagella and cilia are evolutionarily conserved cellular organelles. Abnormal formation or motility of these organelles in humans causes several syndromic diseases termed ciliopathies. The central component of flagella and cilia is the axoneme that is composed of the ‘9+2’ microtubule arrangement, dynein arms, radial spokes, and the Nexin-Dynein Regulatory Complex (N-DRC). The N-DRC is localized between doublet microtubules and has been extensively studied in the unicellular flagellate *Chlamydomonas*. Recently, it has been reported that TCTE1 (DRC5), a component of the N-DRC, is essential for proper sperm motility and male fertility in mice. Further, TCTE1 has been shown to interact with FBXL13 (DRC6) and DRC7; however, functional roles of FBXL13 and DRC7 in mammals have not been elucidated. Here we show that *Fbxl13* and *Drc7* expression are testes-enriched in mice. Although *Fbxl13* knockout (KO) mice did not show any obvious phenotypes, *Drc7* KO male mice were infertile due to their short immotile spermatozoa. In *Drc7* KO spermatids, the axoneme is disorganized and the ‘9+2’ microtubule arrangement was difficult to detect. Further, other N-DRC components fail to incorporate into the flagellum without DRC7. These results indicate that *Drc7*, but not *Fbxl13*, is essential for the correct assembly of the N-DRC and flagella.

## Author summary

In recent years, almost one in six couples face infertility, and nearly 50% of cases are attributed to male factors. It has been shown that approximately 15% of male infertility is caused by genetic factors. The conditions of male infertility mainly include spermatozoa with abnormal morphology (teratozoospermia), reduced sperm motility (asthenozoospermia), and no or low sperm count (azoospermia). Multiple morphological abnormalities of the sperm flagella (MMAF) are characterized as asthenoteratozoospermia, which is a condition with abnormal sperm tail morphology, including absent, coiled, bent, or short tails. Sperm tails are formed during spermiogenesis; however, the mechanism that govern tail formation remains unclear. Here we mutated *Fbxl13* and *Drc7*, two genes with strong expression in mouse testis and which have been shown to be important for flagellum formation and regulation in other systems. Deletion of *Drc7* leads to aberrant tail formation in mouse spermatozoa that phenocopies patients with MMAF, while deletion of *Fbxl13* has no observable effect on sperm function. Our results identified DRC7 as an important factor for sperm flagellum formation.

## Introduction

**Fig 1:**
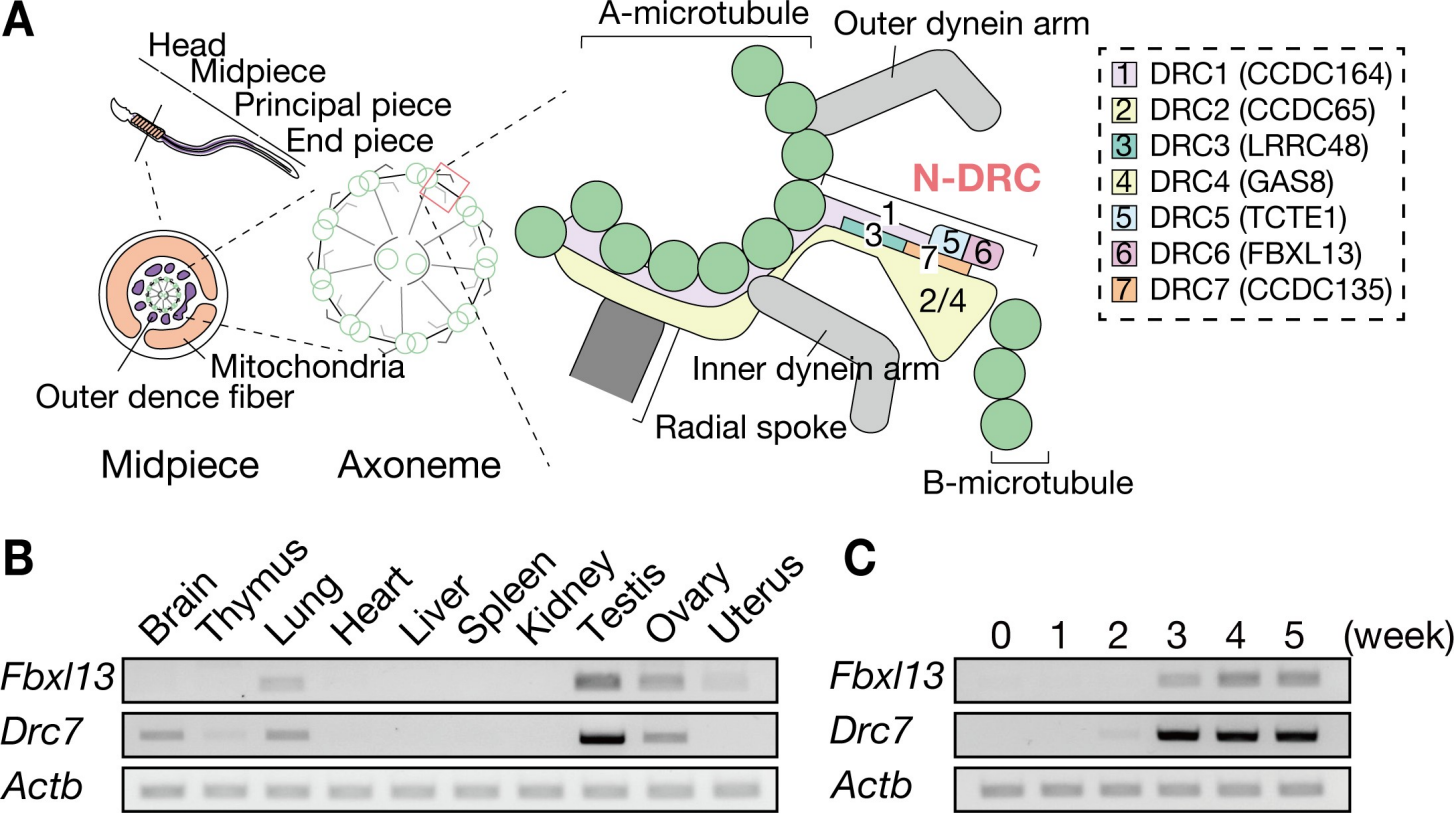
*Fbxl13* and *Drc7* are evolutionarily conserved and testis-enriched genes. (A) Model of DRC localization, based on *Chlamydomonas* [13,14]. The numbers shown in the N-DRC correspond to each DRC component. FBXL13 (DRC6) and DRC7 are located next to TCTE1 (DRC5). (B) The expression of mouse *Fbxl13* and *Drc7* was examined by RT-PCR using RNA isolated from various organs. Both *Fbxl13* and *Drc7* are testis-enriched with weak expression detected in other tissues. *Actb* was used as a loading control. (C) The expression of *Fbxl13* and *Drc7* was examined by RT-PCR using RNA isolated from testis at various postnatal days. *Actb* was used as a loading control.

Cilia and flagella are evolutionarily-conserved microtubule-based organelles extending from the surface of many cell types and are used for sensing and motility [1–3]. Defects in the formation or motility of these organelles are associated with human diseases such as impaired mucociliary clearance and recurrent respiratory infections, which is called primary ciliary dyskinesia (PCD) [4]. The central component of motile cilia and flagella is the axoneme, the ‘9+2’ microtubule arrangement that consists of a central pair (CP) of two singlet microtubules surrounded by nine outer microtubule doublets (Fig 1A) [5,6]. Each doublet microtubule is composed of an A-tubule and a B-tubule. In addition to the microtubule arrangement, the axoneme contains several accessory structures such as inner dynein arms (IDAs), outer dynein arms (ODAs), radial spokes (RSs), and the Nexin-Dynein Regulatory Complex (N-DRC). The IDAs and ODAs are motor complexes attached to the A-tubule and drive axoneme beating by sliding the neighboring doublet microtubules in an ATP dependent manner [7,8]. The RSs extend from the doublet microtubules towards the CP and mediate signal transduction between the CP and the dynein arms [9].

The N-DRC is a large complex that links the A-tubule of one doublet to the B-tubule of the adjacent doublet [10–12]. In the unicellular flagellate *Chlamydomonas reinhardtii*, 11 distinct subunits that compose the N-DRC have been identified [10,13,14]. Further studies using *Chlamydomonas* mutants revealed that the N-DRC plays critical roles in regulating flagellar motility [15–17]. Involvement of N-DRC in regulating flagellar motility has also been clarified in the flagellate protozoa *Trypaosoma brucei* [18–20]. The N-DRC components are conserved evolutionarily from *Chlamydomonas* and *Trypanosoma* to humans and several studies have shown a link between DRC disruptions and ciliary motility defects in vertebrates. For example, *gas8 (drc4*) knockdown leads to impaired cilia motility in zebrafish embryos [21]. Further, mutations in *CCDC164* (*DRC1*), *CCDC65* (*DRC2*), and *GAS8* have been found in PCD patients [22–24]. In mouse models, mutations in *Gas8* and *Lrrc48* (*Drc3*) phenocopy PCD patients [25,26].

In addition to ciliary motility defects, PCD patients can exhibit infertility due to abnormal flagellar movement of the spermatozoa. Although detailed morphology and motility of the spermatozoa were not described, an asthenozoospermia patient with a mutation in *GAS8* was reported [27]. In mice, it was reported that *Lrrc48* mutant males were infertile in addition to displaying abnormal ciliary motility [26]. Further, *Iqcg* (*Drc9*) KO male mice were infertile due to severe defects in flagellar formation and immotility of spermatozoa [28]. Recently, we reported that *Tcte1* (*Drc5*) KO male mice were infertile due to defective sperm motility [29]. We showed that TCTE1 interacts with DRC3, FBXL13 (DRC6), and DRC7 in cultured cell [29], but functional roles of FBXL13 and DRC7 in mammals have not been elucidated. In *Drosophila melanogaster*, it has been reported that insertional mutations of CG34110 (ortholog of mouse *Drc7*) affect sperm movement into the female storage receptacle, suggesting that DRC7 may regulate flagellar motility [30]. In this report, we knocked out *Fbxl13* and *Drc7* and analyzed their function in mouse fertility.

## Results

### *Fbxl13* and *Drc7* are evolutionarily conserved and testis-enriched genes

*Fbxl13* and *Drc7* are evolutionarily conserved genes in many eukaryotes, including *Chlamydomonas*, *Trypanosoma*, mouse, and human (S1 and S2 Figs, S1 Table). Localization of each N-DRC component has been analyzed with biochemical and cryo-electron tomography analyses of *Chlamydomonas* wild-type and mutant axonemes (Fig 1A) [13,14]. To investigate spatial expression of *Fbxl13* and *Drc7* in mice, we conducted multi-tissue RT-PCR using cDNA obtained from adult mice. *Fbxl13* is expressed strongly in testis with weaker signals detected in lung, ovary, and uterus. *Drc7* is also expressed abundantly in testis with weak expression in brain, thymus, lung and ovary (Fig 1B), suggesting that *Fbxl13* and *Drc7* may have some roles in spermatogenesis. In mice, the first wave of spermatogenesis occurs within the first 35 days of postnatal development. [31]. To determine which stage of spermatogenesis *Fbxl13* and *Drc7* start to express, we conducted RT-PCR using cDNA obtained from postnatal testis. Both genes were strongly expressed from three weeks around when spermiogenesis occurs (Fig 1C). These results suggest that *Fbxl13* and *Drc7* function during spermiogenesis when flagellum elongation and head morphogenesis occur. FBXL13 or DRC7 localization in the mouse testis and spermatozoa was not analyzed due to the unavailability of effective antibodies against FBXL13 and DRC7. However, in a previous study, DRC7 was detected in the tail fraction of human spermatozoa using proteomic analysis [32]. Further, in *Drosophila*, DRC7 is localized in the entire flagellum of the spermatozoa [30].

### Generation of *Fbxl13* or *Drc7* knockout mice

To uncover the function of *Fbxl13* and *Drc7 in vivo*, we generated *Fbxl13* and *Drc7* single knockout (KO) mice. To generate *Fbxl13* KO mice we mutated the gene in ES cells using the CRISPR/Cas9 system [33] (S3A Fig). Because exon 10–12 overlapped with *Lrrc17*, we designed guide RNAs (gRNA) targeting exon 1 and exon 7. These gRNA sequences were inserted into pX459 plasmids that express humanized CAS9 [33]. The plasmids were co-transfected into EGR-G01 ES cells [34] to excise the region flanked by the two gRNA target sequences. We obtained 5 mutant clones with biallelic large deletions out of 48 clones examined with three clones used for injecting into 8-cell embryos. The chimeric mice obtained with biallelic deletions were mated with wild-type females, and heterozygous mice with a 30983 bp deletion were obtained, suggesting that this gene might not be involved in male fertility. To confirm the function of this gene, homozygous KO mice were obtained by subsequent mating (S3B Fig). We confirmed that mRNA was not detected in the *Fbxl13*^*-/-*^ testis with RT-PCR using primers designed within exon 1 and 20 (S3C Fig). No overt abnormalities were observed in *Fbxl13*^*-/-*^ mice.

For *Drc7* KO mice, we established mutant ES clones (EGR-G101) [34] using the targeting vector obtained from Knockout Mouse Project (KOMP). Ten clones out of 48 clones were targeted correctly and three clones were injected into 8-cell embryos. Chimeric mice were mated with wild-type females. The mutant mice obtained were mated with CAG-Cre transgenic mice to delete the region flanked by loxP sites (tm1b allele) (S3D Fig). Homozygous KO mice were obtained by subsequently mating of tm1b heterozygous mice (S3E Fig). We confirmed that mRNA was not detected in the *Drc7*^*-/-*^ testis with RT-PCR using primers designed within Exon 15 and 18 (S3F Fig). No overt abnormalities were observed in *Drc7*^*-/-*^ mice.

### *Fbxl13* is not required for male fertility

In order to analyze the fertility of *Fbxl13*^*-/-*^ male mice, individual wild-type or homozygous males were caged with wild-type females for two months. Homozygous males sired a comparable number of pups to the wild-type males (S4A Fig). To investigate if there were any subtle abnormalities not affecting male fertility, we examined spermatogenesis with periodic acid-Schiff (PAS) staining of testis sections, however, no abnormalities were observed in spermatogenesis of *Fbxl13* KO testis (S4B Fig). The morphology of KO spermatozoa obtained from the cauda epididymis was also normal (S4C Fig). Further, we examined sperm motility using a Computer Assisted Sperm Analyzer (CASA). No significant differences were found in both motility rates and progressive motility rates (S4D Fig). In addition, velocity parameters such as average path velocity (VAP), straight line velocity (VSL), and curvilinear velocity (VCL) [35] were normal in *Fbxl13* KO mice (S4D Fig). Taken together, these results indicate that *Fbxl13* is not essential for male fertility, sperm formation, or sperm motility.

### *Fbxl13* is not required for ciliogenesis in the trachea

Although no overt abnormalities were observed in *Fbxl13*^*-/-*^ mice, *Fbxl13* expression was detected in tissues other than testis. To examine the role of FBXL13 in other ciliated tissue, multicilia of tracheal epithelia were analyzed as *Fbxl13* expression was detected in the lungs (Fig 1B). No morphological abnormality was observed by scanning electron microscopy (S5A Fig) and the ‘9+2’ arrangement of microtubules was observed in the *Fbxl13*^*-/-*^ mice (S5B Fig). These data indicate that motile cilia can form in the *Fbxl13*^*-/-*^ trachea.

### *Drc7* is required for male fertility, head morphogenesis, and sperm flagellum formation

**Fig 2:**
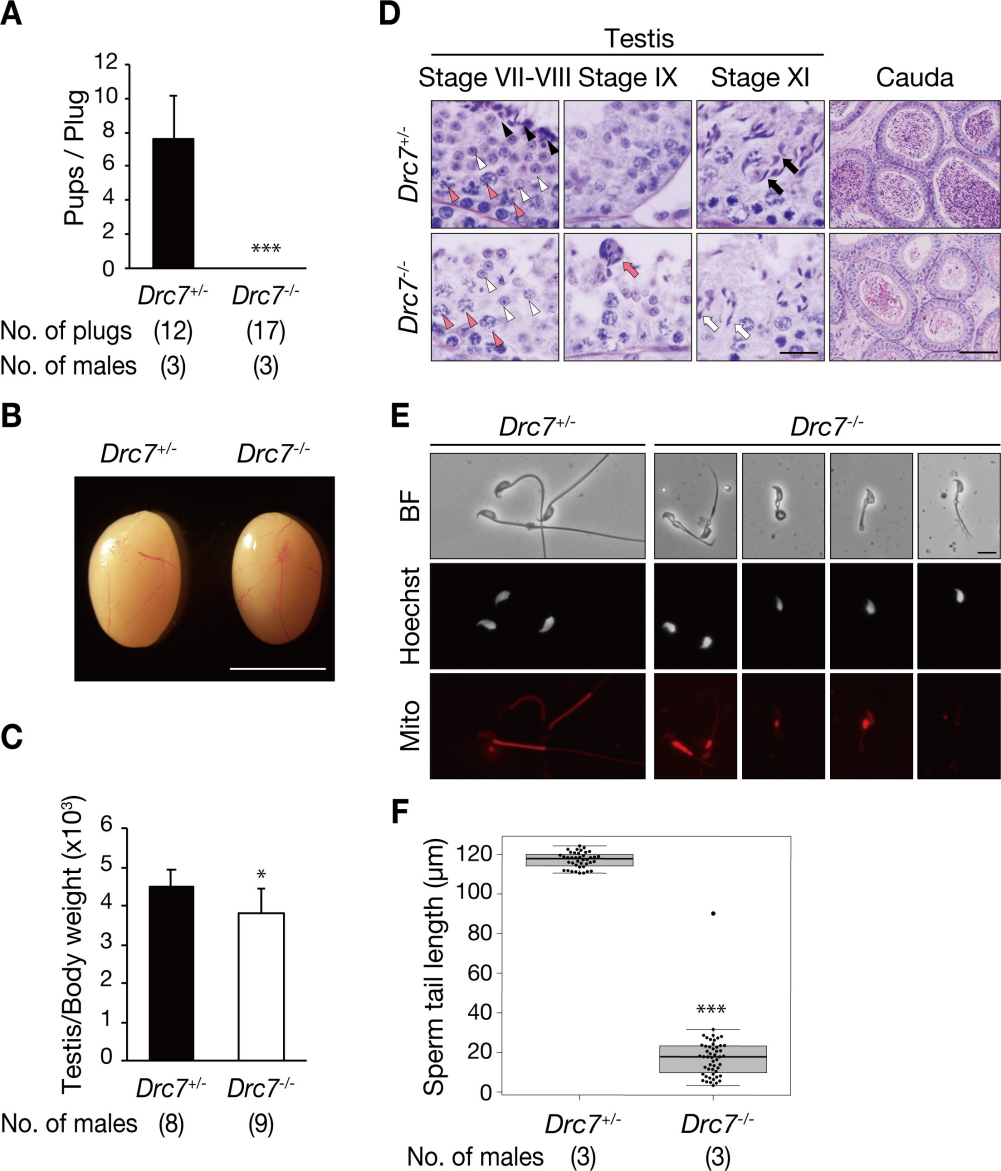
*Drc7* is required for male fertility, head morphogenesis, and sperm flagellum formation. (A) *Drc7*^*+/-*^ or *Drc7*^*-/-*^ males were mated with wild-type females and the number of pups born per plug is reported. Although 17 plugs were observed, *Drc7*^*-/-*^ male mice did not sire offspring. Error bars represent S.D. ***p* *< 0.001 (unpaired Student's t-test). (B) Testis morphology and (C) testis/body weight of *Drc7*^*+/-*^ and *Drc7*^*-/-*^ adult mice. Error bars represent S.D. *p* *< 0.05 (unpaired Student's t-test). Scale bar, 5 mm. (D) PAS staining of seminiferous tubules and cauda epididymis of adult mice. In stage VII-VIII, normal spermatocytes (red arrowhead) and round spermatids (white arrowhead) were observed in both *Drc7*^*+/-*^ and *Drc7*^*-/-*^ testis; however, elongated spermatids (black arrowhead) were hardly detected in *Drc7*^*-/-*^. In stage IX, elongated spermatids were released in *Drc7*^*+/-*^ but they were retained in *Drc7*^*-/-*^ (red arrow). Stage XI step 11 spermatids of *Drc7*^*+/-*^ mice had normal, hook shaped heads (black arrow) whereas *Drc7* KO step 11 spermatids have abnormal “club-shaped” heads (white arrow). In cauda epididymis, spermatozoa were hardly seen in *Drc7*^*-/-*^ mice. Scale bar, 50 μm. (E) Observation of spermatozoa extracted from cauda epididymis. Sperm mitochondria (midpiece) is stained with Mitotracker (red). Spermatozoa from *Drc7*^*-/-*^ mice have abnormal head shapes, short tails, disrupted midpieces, and are immotile. Hoechst (white), nucleus. Scale bar, 10 μm. (F) The measurement of sperm tail length. *Drc7* KO spermatozoa exhibit shorter tails. ***p* *< 0.001 (unpaired Student's t-test).

To analyze the fertility of *Drc7*^*-/-*^ male mice, individual heterozygous or homozygous males were caged with wild-type females for two months. Although the formation of plugs was observed, homozygous males failed to sire any pups (Fig 2A). To investigate the causes of infertility in *Drc7*^*-/-*^ male mice, we observed testis morphology and found that *Drc7*^*-/-*^ testes were slightly smaller than those of *Drc7*^*+/-*^ (Fig 2B and 2C). In testis sections with PAS staining, spermatogonia, spermatocytes, and round spermatids were observed in homozygous mice; however, elongated spermatids were hardly detected (Fig 2D, Stage VII-VIII). Further, elongated spermatids were not released and abnormally retained in stage IX seminiferous tubules of *Drc7*^*-/-*^ mice (Fig 2D). In addition, abnormal head shapes were observed in stage XI (step 11) of *Drc7*^*-/-*^ testis (Fig 2D).

PAS staining of cauda epididymis sections indicated that the control tubules were filled with mature spermatozoa, but spermatozoa were hardly observed in the *Drc7*^*-/-*^ epididymis (Fig 2D, Cauda). When we observed the spermatozoa obtained from the *Drc7*^*-/-*^ cauda epididymis, they exhibited significantly shorter tails and abnormal head shapes (Fig 2E and 2F), and were immotile (number of motile spermatozoa = 0 out of 101 spermatozoa observed, number of males = 4) (S1 and S2 Movies). Further, aberrant localization or loss of mitochondria were observed with Mitotracker staining (Fig 2E). These results indicate that *Drc7* is necessary for spermiogenesis, especially for flagellum formation and head morphogenesis.

### Other N-DRC components in *Drc7* KO spermatozoa

**Fig 3:**
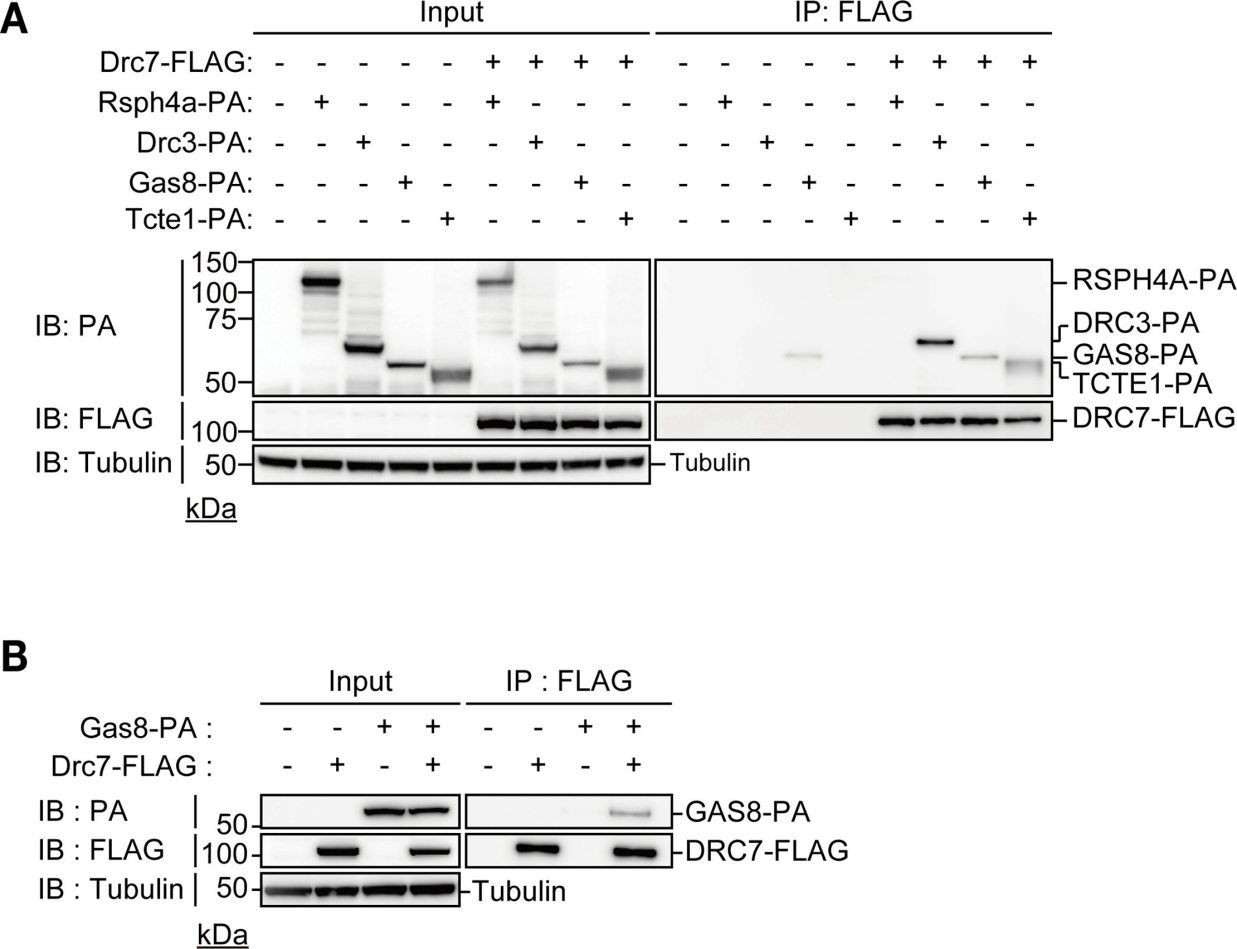
Analysis of DRC7 interaction with other N-DRC. (A) Analysis of DRC7 interaction with other N-DRC components using HEK293T cells. DRC7 can bind to DRC3 and TCTE1 in HEK293T cells. α-Tubulin was used as a loading control. (B) DRC7 can bind to GAS8 in HEK293T cells. α-Tubulin was used as a loading control.

We investigated the interaction of N-DRC components. We focused on DRC3, GAS8, and TCTE1 because these DRCs were shown to be localized adjacent to DRC7 in *Chlamydomonas* flagella (Fig 1A) [13–15]. We co-expressed FLAG-tagged mouse DRC7 and PA-tagged mouse DRC3, GAS8, or TCTE1 in HEK293T cells and confirmed that mouse DRC7 interacts with mouse DRC3 and TCTE1 but not RSPH4A, a radial spoke protein, using immunoprecipitation analysis (Fig 3A). Because PA-tagged GAS8 bound non-specifically to a mouse monoclonal anti-FLAG antibody (Fig 3A), we utilized a rabbit polyclonal antibody for FLAG for immunoprecipitation and the results showed that DRC7 can also interact with GAS8 (Fig 3B)

**Fig 4:**
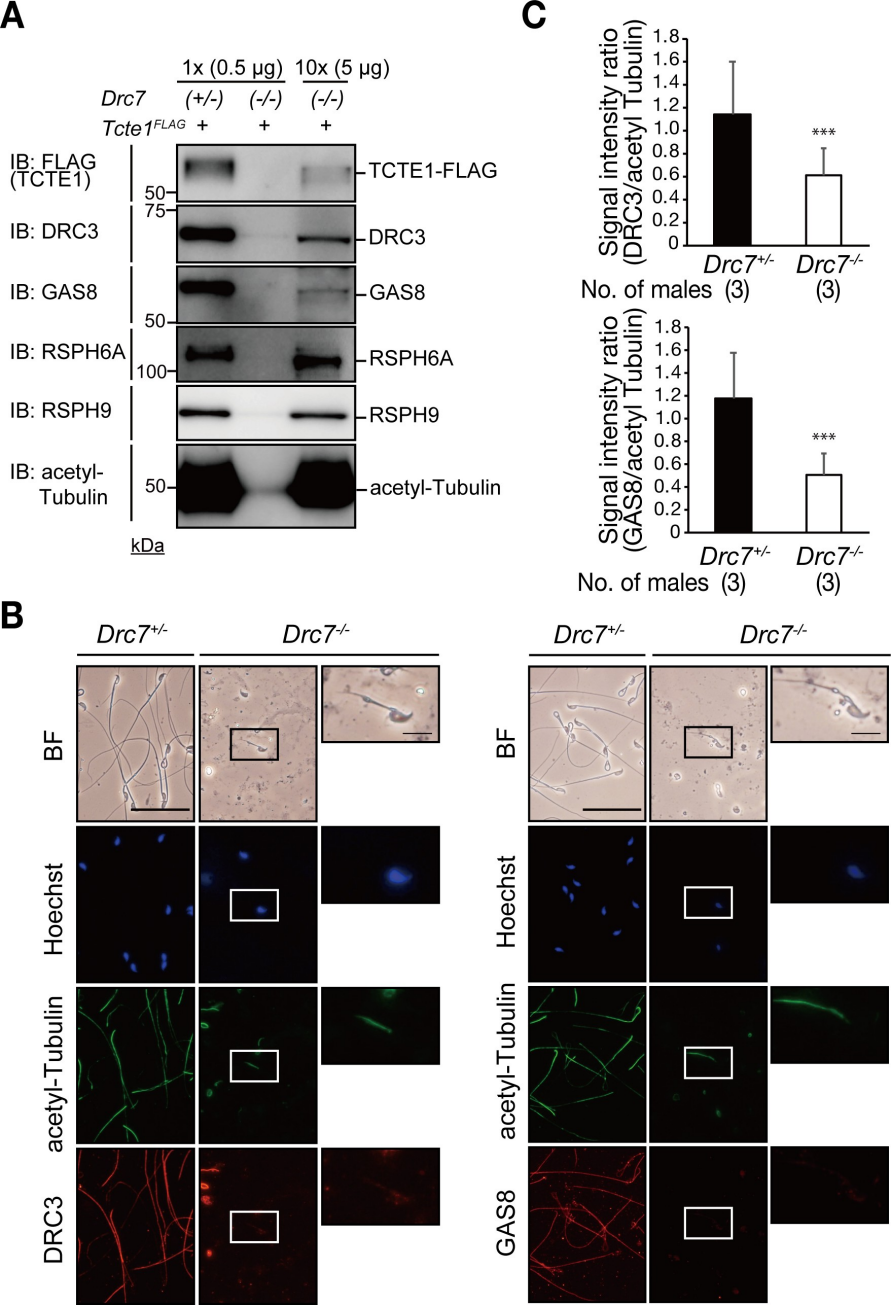
The structure of N-DRC is disrupted in *Drc7* KO spermatozoa. (A) Presence of other N-DRC components in spermatozoa. *Drc7* KO spermatozoa had decreased levels of DRC3, GAS8 and TCTE1. In contrast, the signal of RSPH6A and RSPH9 were detected at comparable levels. (B) Immunostaining of DRC3 (left) and GAS8 (right) in spermatozoa. Enlarged pictures of *Drc7* KO spermatozoa are shown in right panel. Hoechst (blue), nucleus; acetylated-tubulin (green); DRC3 or GAS8 (red). Scale bar in upper, 50 μm, below, 10 μm. (C) Signal intensity ratio of DRC3/acetylated tubulin (upper) and GAS8/acetylated tubulin (lower) shown in (B). The signal of DRC3 and GAS8 were significantly weaker in *Drc7* KO spermatozoa. Error bars represent S.D. ***p* *< 0.001 (unpaired Student's t-test).

Next, we confirmed if the localization of other N-DRC components were disrupted in the short flagellum of *Drc7* KO spermatozoa. Because we could not obtain an effective TCTE1 antibody, we crossed *Drc7* mutant mice with a mouse line that has a FLAG sequence knocked into the 3’ end of the *Tcte1* locus [29]. With Western blot analysis using FLAG antibody, we found that the amount of TCTE1-FLAG decreased in the *Drc7* KO spermatozoa (Fig 4A). In addition, the amount of DRC3 and GAS8 decreased as well; however, the decreased levels could be due to the short tails of *Drc7* KO spermatozoa. To normalize the amount of loaded protein we utilize acetylated tubulin signal from Western blot analysis. Ten times the volume of protein lysate was loaded and the results showed that the amount of TCTE1, DRC3 and GAS8 decreased in *Drc7* KO spermatozoa. In contrast, the signal of RSPH6A and RSPH9, proteins localized in the radial spokes, was comparable to control. We also performed immunofluorescence analysis to further detect the localization of DRC3 and GAS8. Both DRC3 and GAS8 were localized in the sperm flagellum and their fluorescence intensities were significantly lower in *Drc7* KO spermatozoa (Fig 4B and 4C). These results suggest that the structure of the N-DRC is disrupted because of the absence of DRC7.

### Axoneme formation and manchette removal is impaired in *Drc7*^*-/-*^ testis

**Fig 5:**
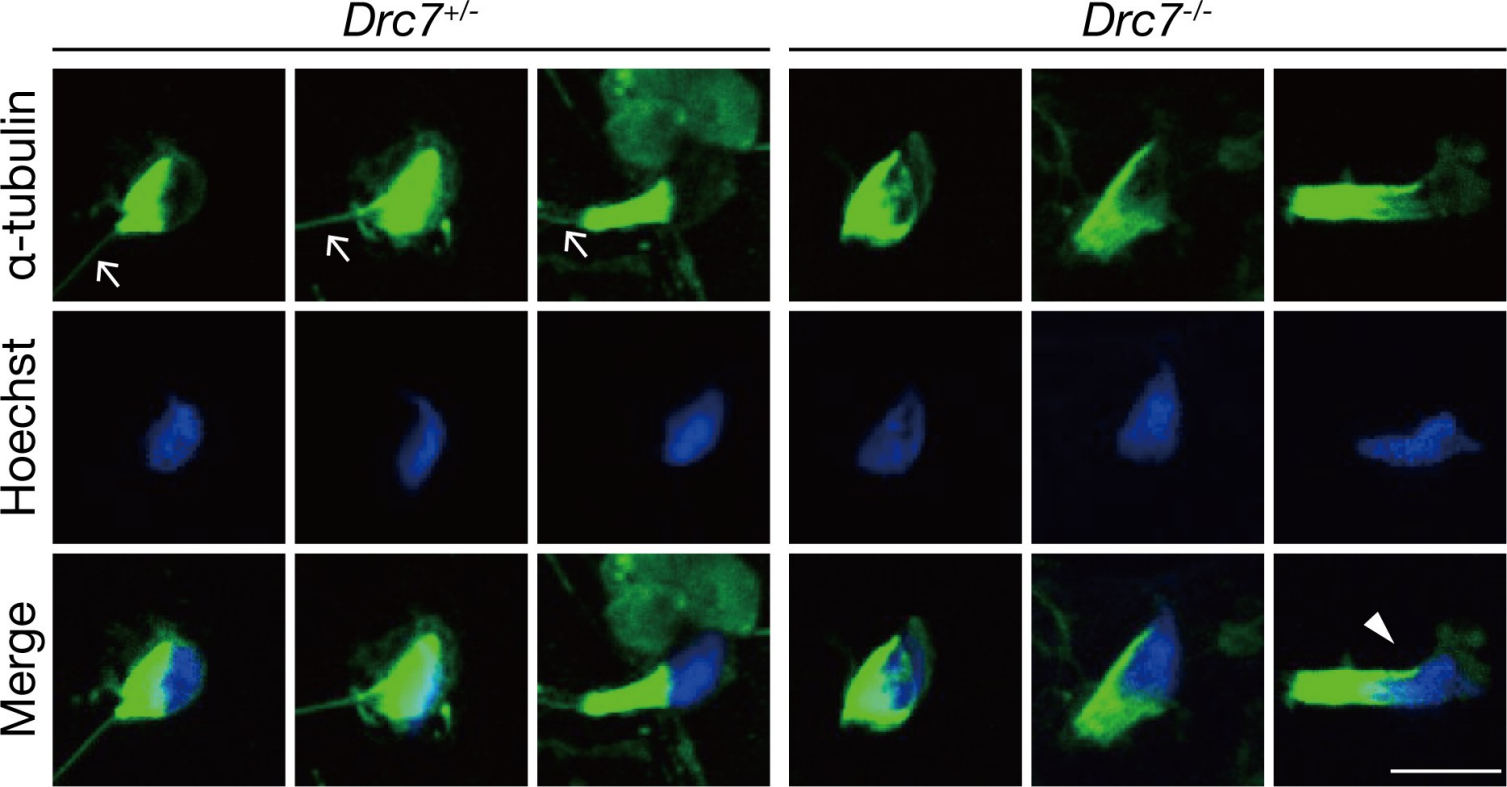
Axoneme formation and manchette removal is impaired in *Drc7*^*-/-*^ testis. Immunofluorescence analysis of spermatids from *Drc7*^*+/-*^ and *Drc7*^*-/-*^ mice. α-tubulin (green) stains both manchette and axoneme. Elongating spermatids are shown progressively from left to right. Arrows indicate axoneme. Axoneme formation is severely impaired in *Drc7* KO spermatids. An arrowhead indicates abnormal caudal movement of the manchette. Hoechst (blue), nucleus. Scale bar, 10 μm.

To further analyze the causes of abnormal sperm morphology in *Drc7*^*-/-*^ mice, we observed microtubule structures that play important roles in tail elongation and head morphogenesis during spermiogenesis (Fig 5). At the early steps of spermiogenesis, the axoneme composed of microtubules elongates and a transient microtubule structure called the manchette is assembled [36]. The manchette is involved in sperm head elongation, and abnormal head morphology is often related to defects in the formation, function, and movement of the manchette [37–40]. To observe the axoneme and manchette, we performed immunofluorescence using an anti-α-tubulin antibody on spermatogenic cells obtained from seminiferous tubules. From control mice, the elongating axoneme was observed in all steps. In contrast, we could not find the axoneme in *Drc7*^*-/-*^ spermatids, suggesting that axoneme formation is severely impaired in *Drc7* KO spermatids. The formation of the manchette was comparable between the control and *Drc7* KO spermatids in the early step of spermiogenesis, but in later steps, the movement of the manchette was aberrant in *Drc7* KO mice. In the control, the manchette tightened around the nucleus correctly as it moves caudally. On the other hand, in the *Drc7* KO mice, the caudal movement of the manchette was delayed, which leads to the contraction of the manchette at an unusual location and subsequent formation of the “club-shaped” head. These results indicate that the failure of axoneme elongation precedes the formation of abnormal sperm heads in the *Drc7* KO spermatids.

### Ultrastructural analysis of the testis in *Drc7*^*-/-*^ mice

**Fig 6:**
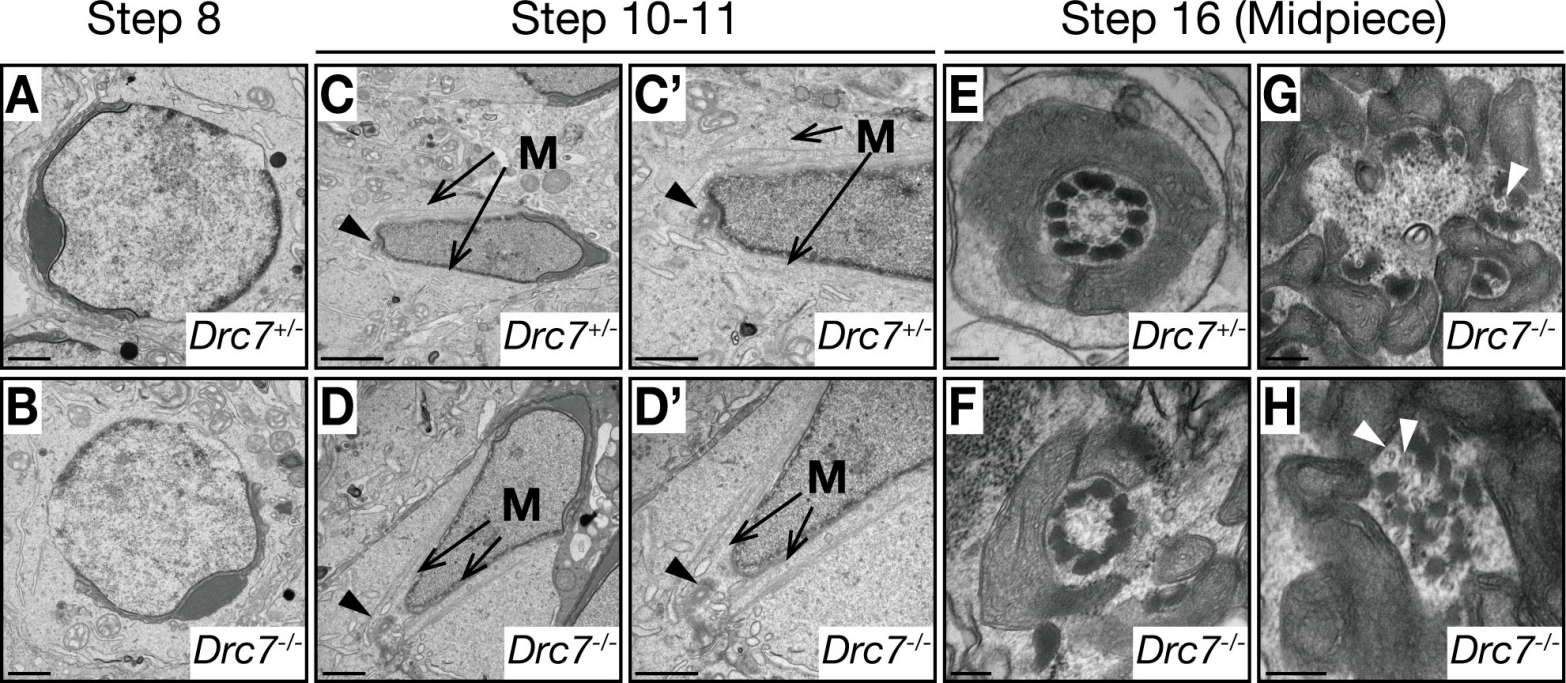
Ultrastructural analysis of *Drc7*^*-/-*^ testis. (A) and (B) Step 8 spermatids. No difference was observed between control and KO spermatids. Scale bar, 1 μm. (C) and (D) Step 10–11 spermatids. Both control and KO spermatids show a normal manchette structure (shown as “M”). Basal body (black arrowheads) were localized to the posterior region of the nucleus in both control and KO spermatids. Enlarged pictures are shown in (C’) and (D’). Scale bar in (C) is 2 μm and scale bars in (C’), (D), and (D’) are 1 μm. (E)—(H) Cross section of the midpiece of step 16 spermatids. Spermatids from *Drc7*^*+/-*^ mice show normal arrangement of mitochondria, outer dense fibers, and microtubules (E). In *Drc7* KO spermatids, ‘9+2’ arrangement of microtubules was absent (F) or disorganized (G) and (H). White arrowheads indicate microtubule-like structures. Scale bar, 200 nm.

To investigate axoneme elongation in *Drc7*^*-/-*^ mice in more detail, we observed the testis from *Drc7*^*+/-*^ and *Drc7*^*-/-*^ mice using transmission electron microscopy (TEM). Consistent with the PAS staining, normal round spermatids (step 8) were observed in *Drc7*^*-/-*^ testis (Fig 6A and 6B). The manchette formation in elongating *Drc7* KO spermatids was also comparable with that of *Drc7* heterozygous spermatids (Fig 6C and 6D). Further, in step 10–11 elongating spermatids, the basal body, a structure for axoneme extension, was correctly located on the posterior side of the nucleus in the *Drc7* KO spermatids (Fig 6C’ and 6D’). However, it was difficult to find the flagella in the *Drc7*^*-/-*^ testis (Fig 6E–6H). In the rare *Drc7* KO flagellum found, the arrangement of mitochondria and outer dense fibers were abnormal, and the inner ‘9+2’ arrangement of microtubules were missing (Fig 6F). Very rarely in these *Drc7* KO flagellum, scattered microtubule-like structures were found (Fig 6G and 6H), which is consistent with the detection of acetylated tubulin in the mature *Drc7* KO spermatozoa (Figs 4B, 4D and 5). These results suggest that the basal body can be located correctly, but flagellum elongation was impaired because the axoneme structure was disrupted in the *Drc7*^*-/-*^ mice.

### *Drc7* is not required for ciliogenesis in the brain and trachea

*Drc7* expression was detected in tissues other than testis, however no overt abnormalities were observed in *Drc7*^*-/-*^ mice. To examine the role of DRC7 in other ciliated tissues, multicilia of ependymal and tracheal epithelia were observed because *Drc7* expression was detected in these tissues. No morphological abnormalities were observed by scanning electron microscopy (S5A and S6A Figs) and the ‘9+2’ arrangement of microtubules was observed in the *Drc7*^*-/-*^ mice (S5B and S6B Figs). In addition, cilia motility was not impaired in ependymal cilia (S3 and S4 Movie). These results indicate that motile cilia can be formed in the *Drc7*^*-/-*^ brain and trachea.

### Normal pups are obtained from *Drc7* KO spermatozoa with ICSI

**Fig 7:**
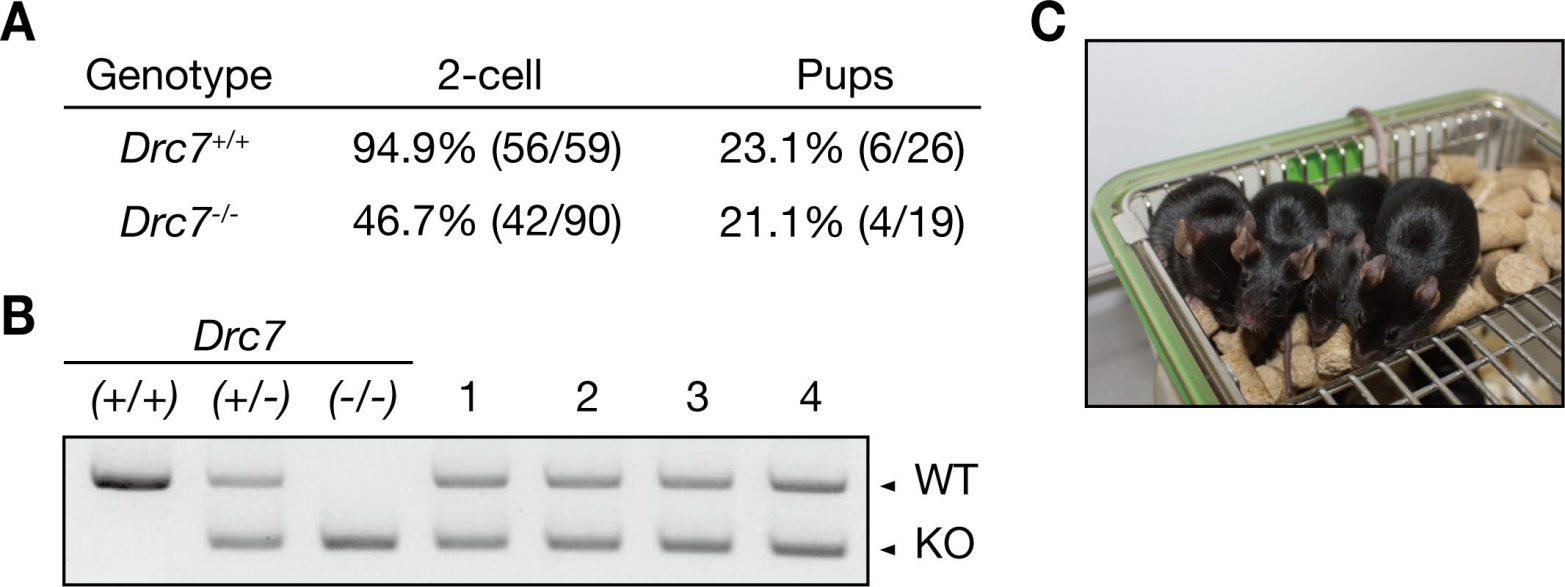
Normal pups were obtained from *Drc7* KO spermatozoa with ICSI. (A) Development of ICSI embryos. Oocytes injected with *Drc7* KO spermatozoa developed to the 2-cell stage and pups were obtained after embryo transfer. (B) Genotyping of obtained pups. Four pups were derived from *Drc7* KO spermatozoa. (C) Pups derived from *Drc7* KO spermatozoa.

To investigate if *Drc7* KO sperm nuclei can produce viable pups despite impaired spermiogenesis, we conducted intracytoplasmic sperm injection (ICSI). Ninety wild-type oocytes were injected with *Drc7* KO spermatozoa obtained from cauda epididymis and 42 of them were developed to the 2-cell stage (Fig 7A). These embryos were transplanted into pseudopregnant female mice and 4 heterozygous pups were obtained (Fig 7B). These pups grew normally (Fig 7C). These results showed that *Drc7* KO sperm nuclei could activate eggs and produce viable pups, and the infertility phenotype seen in *Drc7* KO males could be rescued with ICSI.

## Discussion

*Fbxl13* and *Drc7* are evolutionarily conserved genes that are present in nearly all eukaryotes including *Chlamydomonas*, *Trypanosoma*, flies, mice, and humans. In mice, we showed that these genes are testis-enriched and start to express during the haploid phase of spermatogenesis, when several flagellar proteins are translated to form the axoneme [41]. Despite showing testis-enriched expression, *Fbxl13* is not necessary for male fertility, sperm formation, or sperm motility. According to the previous report in *Chlamydomonas*, DRC6 (FBXL13 ortholog) is located in the most distal region adjacent to the B-tubule [10,13]; therefore, FBXL13 may not play critical roles compared to proteins localized at the base of the N-DRC. It is also possible that there is either a paralogue or an analog that replaces FBXL13 in mouse flagella. In *Chlamydomonas*, a structural paralogue (FAP43) of a flagella protein (FAP244) has been shown to replace FAP244 [42]. BLAST searches against *Chlamydomonas* DRC6 in mice reveal that FBXL4 and FBXL7 are potential orthologs. These two proteins may replace the function of FBXL13.

In contrast, in the absence of DRC7, male mice were infertile due to flagellar malformation. This phenotype is different from CG34110 (ortholog of mouse *Drc7*) mutants in *Drosophila melanogaster* [30], which do not exhibit short flagella but show impaired sperm motility, suggesting that flagellar formation may be slightly different in *Drosophila* and mice. For example, in *Drosophila*, intraflagellar transport (IFT) is not required for the assembly of flagella [43,44], whereas it is reported that IFT proteins are required for sperm flagellum formation in mouse [45]. Immunostaining of the axoneme during spermiogenesis suggests that the axoneme formation is severely impaired in *Drc7* KO spermatids. Consistent with this result, we could not detect any organized ‘9+2’ microtubule arrangement in the *Drc7* KO testis with electron microscopy. Mitochondrial sheaths and other accessory structures were also misshapen. Because other N-DRC components were reduced in the *Drc7* KO spermatozoa, DRC7 may stabilize the N-DRC, which is essential for proper axoneme elongation. A disorganized axonemal structure could lead to impaired IFT transport on the B-tubule of the outer-doublets, which may cause flagellar shortening in the *Drc7* KO spermatozoa. In contrast, in the *Tcte1* KO mice, the axoneme structure was normal, but their sperm motility was impaired [29]. In *Chlamydomonas*, it is indicated that DRC5 (TCTE1 orthologue) is localized to a more distal region from the A-tubule than DRC7, suggesting that DRC7 is essential to localize TCTE1 in the correct place, and that TCTE1 regulates flagellar motility. This is supported by the observation that TCTE1 decreased in *Drc7* KO spermatozoa, but DRC7 was detected in the *Tcte1* KO spermatozoa [29].

Despite severe phenotypes found in spermatozoa, no defects were recognized in the ependymal and tracheal cilia of the *Drc7* KO mice. A recent study has shown that *drc7* mutant *Chlamydomonas* does not display an obvious motility defect, which is consistent with no obvious ciliary abnormalities found in the *Drc7* KO mice in this study. As sperm flagellum has accessory structures such as outer dense fibers, fibrous sheaths, and mitochondrial sheaths, sperm flagellum formation may be different from that of cilia and *Chlamydomonas* flagella. This idea is supported by several KO mouse lines that show normal ciliary formation, but exhibit impaired sperm flagellum formation [46]. Alternatively, it is possible that other proteins compensate the function of DRC7 in the ependymal and tracheal cilia. Proteins used for axonemal formation are known to be different between cilia and sperm flagellum [47].

In addition to flagellar deformation, abnormal sperm head morphology was observed in *Drc7* KO mice. Because manchette movement is abnormal, it is likely that tightening of the sperm heads by the manchette occurs at an incorrect place is causing the abnormal morphology. Since flagellar deformation precedes the formation of the misshapen heads in *Drc7* KO testis, impaired manchette movement may be a secondary effect of abnormal flagellar formation. As it is suggested in the previous study [48], proper flagellar formation may be required to remove the manchette properly. It is noteworthy to mention that mutations of axonemal proteins often exhibit both abnormal flagellar formation and misshapen heads in mice [37,39,48].

In summary, our results indicate that *Drc7* is essential for flagellar formation and male fertility. The phenotype of *Drc7* KO mice is similar to human reproduction disorders called multiple morphological abnormalities of the flagellum (MMAF). The genes identified as associated with MMAF thus far account for only one-third of cases with the remaining two-thirds having unknown etiology [49]. By clarifying the interactions between the N-DRC proteins, this may give us a better understanding of the mechanism involved in sperm flagellar formation and may lead us to uncover the remaining causative genes of MMAF.

## Materials and methods

### Ethics statement

All animal experiments were conducted in accordance with the guidelines of “Animal experiment rules” established by the Research Institute for Microbial Diseases, Osaka University, and were approved by the Animal Care and Use Committee of the Research Institute for Microbial Diseases, Osaka University (#Biken-AP-H30-01).

### Animals

All mice used in this study were purchased from CLEA Japan (Tokyo, Japan) or Japan SLC (Shizuoka, Japan). All mice were maintained under specific-pathogen-free conditions with *ad libitum* feeding. The mouse line with FLAG knocked into the 3’ end of the *Tcte1* locus (*Tcte1^tm2(Maik)^*) was maintained in our laboratory [29].

### Isolation of RNA and RT-PCR

RNA was isolated and purified from multiple adult tissues of C57BL/6N mice at different ages with TRIzol (15596018, Thermo Fisher Scientific). Reverse transcription was performed using purified RNA and the SuperScript III first-strand synthesis system (18080051, Thermo Fisher Scientific). PCR was carried out using KOD Fx neo (KFX-201, TOYOBO, Osaka, Japan). The primers for each gene are listed in S2 Table.

### Generation of *Fbxl13*-deficient mice

*Fbxl13* large deletion (LD) KO mice were generated as previously described [33]. We designed two gRNAs that recognize either exon 1 or exon 7 to avoid the cleavage of nearby genes (S2A Fig) and inserted the sequences into the pX459 V2.0 plasmid (#62988, Addgene, Cambridge, MA, USA). EGR-G01 embryonic stem (ES) cells [34] were co-transfected with 1.0 μg of two gRNAs inserted vectors. Nine ES clones out of 48 had a large deletion of *Fbxl13*. ES cell clones that possessed the mutation were injected into ICR embryos and chimeric blastocysts were transferred into the uteri of pseudopregnant females. Generated chimeric male mice were mated with B6D2F1 female mice to obtain heterozygous KO mice. *Fbxl13* KO mice were maintained by sibling matings.

### Generation of *Drc7*-deficient mice

A targeting vector for *Drc7* (#PG00082_Z_3_D01) was obtained from the KnockOut Mouse Project (KOMP) and electroporated into EGR-G101 ES cells [34] after linearization. Potentially targeted ES cell clones were selected with G418. Correctly targeted ES cell clones and germ-line transmission were determined via PCR using primers listed in S2 Table. Ten ES clones that were correctly targeted were obtained out of 48 clones. Heterozygous KO mice were generated from the ES cells in the same way as *Fbxl13* KO mice.

### Genotyping analysis

PCR was performed using KOD FX neo (KFX-201, TOYOBO). The primers for each gene are listed in S2 Table.

### *In vivo* male fertility check

Each control (wild-type or *Drc7*^*+/-*^) or KO male was caged with 2 or 3 8-week-old B6D2F1 female mice for 2 months. Plugs and the number of sired pups were checked every morning.

### Morphological, histological, and motility analysis of testis and spermatozoa

Testes and epididymides were fixed overnight at 4°C in Bouin’s fluid (16045–1, Polysciences, Inc., Warrington, PA, USA) and embedded in paraffin. Paraffin sections (5 μm) were rehydrated, treated with 1% periodic acid for 20 min at room temperature, and incubated with Schiff's reagent (193–08445, FUJIFILM Wako Pure Chemical, Osaka, Japan) for 20 min at room temperature. The sections were stained with Mayer's haematoxylin solution (131–09665, FUJIFILM Wako Pure Chemical) and observed using a BX-50 microscope (Olympus, Tokyo, Japan).

Cauda epididymal spermatozoa were dispersed in TYH medium [50] or non-capacitating medium [51] for 10 min before the observation of morphology and motility. For mitochondria staining, spermatozoa were dispersed in TYH medium containing 10 nM MitoTracker Red FM (M22425, Thermo Fisher Scientific) and 10 μg/mL of Hoechst 33342 (H3570, Thermo Fisher Scientific). After 10 min incubation at 37°C under 5% CO_2_ in air, spermatozoa were observed using a phase contrast microscope (BX50, Olympus).

### Sperm velocity analysis

Sperm velocity was analyzed as described previously [52]. Cauda epididymal spermatozoa were suspended in TYH medium [50]. Sperm velocity was measured using the CEROS sperm analysis system (software version 12.3; Hamilton Thorne Biosciences, Beverly, MA, USA) at 10 min and 2 h after incubation. More than 200 spermatozoa were analyzed for each male.

### Protein extraction of testis and spermatozoa

Protein samples were extracted from testis and spermatozoa using lysis buffer (6 M urea, 2 M thiourea, and 2% sodium deoxycholate) and centrifuged at 15,300 × *g* for 15 min at room temperature. Obtained supernatants were subjected to SDS-PAGE for immunoblotting.

### Construction of plasmid

cDNA encoding *Drc3*, *Gas8*, *Tcte1*, and *Drc7* were amplified from mouse testis (C57BL/6N) and cloned into FLAG-tagged (C-terminus) or PA-tagged (C-terminus) pCAG vectors that contain the CAG promoter and a rabbit globin poly(A) signal [53]. PA-tagged *Rsph4a* vector was described previously [48]. The primers used to amplify each gene are listed in S2 Table.

### Cell culture and transfection

HEK293T cells were cultured in DMEM (11995–065, Thermo Fisher Scientific) supplemented with 10% fetal bovine serum (S1560, Biowest, Nuaillé, France) and 1% Penicillin-Streptomycin-Glutamine (10378–016, Thermo Fisher Scientific) at 37°C under 5% CO_2_ in air. Plasmid DNA were transiently transfected into HEK293T cells [54] and cultured for 48 h.

### Immunoprecipitation

Harvested cells were lysed with a solution containing 1% Triton X-100, 50 mM NaCl, 20 mM Tris-HCl, pH 7.4, Protease Inhibitor Cocktail (25955, Nacalai tesque, Kyoto, Japan), and incubated for 30 min at 4°C. The lysates were centrifuged at 15,300 × *g* for 15 min at 4°C, and the resulting supernatants were incubated for 60 min at 4°C with FLAG (M2 or PM020) conjugated Dynabeads (10009D, Thermo Fisher Scientific). The immune complexes were then washed three times with a solution containing 40 mM Tris-HCl, 150 mM NaCl, 0.1% Triton X-100, and 10% glycerol and eluted with sample buffer with 2-mercaptoethanol before SDS-PAGE and immunoblot analysis.

### Immunoblotting

Proteins obtained from culture cells or tissues were separated by SDS-PAGE under reducing conditions and transferred to PVDF membrane using the Trans Blot Turbo system (Bio-Rad, Foster City, CA, USA). After blocking with 5% skim milk (232100, Becton Dickinson, Cockeysville, MD, USA), the membrane was incubated with primary antibodies overnight at 4°C and then incubated with HRP-conjugated secondary antibodies. Chemiluminescence was detected by Chemi-Lumi One Super (02230, Nacalai tesque) or Chemi-Lumi One Ultra (11644, Nacalai tesque) using Image Quant LAS 4000 mini (GE Healthcare, Buckinghamshire, UK). Antibodies used are listed in S3 Table. Antibody for RSPH6A was generated as previously described [48].

### Immunofluorescence

Immunofluorescence for mature spermatozoa was conducted as described previously [48] with slight modifications. Spermatozoa collected from the cauda epididymis were diluted in PBS, spread onto glass slides, and incubated at 37°C until dry. The samples were fixed with 4% paraformaldehyde in PBS for 20 min and fixed with 100% methanol at -30°C for 3 min. After three 10 min washes with 0.1% Triton X-100 in PBS, the slides were blocked with 5% BSA and 10% goat serum for 1 h at room temperature and incubated with primary antibodies overnight at 4°C. After three 10 min washes with 0.1% Triton X-100 in PBS, the slides were incubated with secondary antibody at room temperature for 90 min and then washed with 0.1% Triton X-100 in PBS three times for 10 min each. To stain the nucleus, the slides were incubated with Hoechst 33342 (H3570, Thermo Fisher Scientific) for 15 min, washed with 0.1% Triton X-100 in PBS three times for 10 min each, and then mounted using Immu-Mount (9990402, Thermo Fisher Scientific). Slides were observed with a BX-53 microscope (Olympus).

Immunofluorescence for manchette was conducted as described previously [48] with slight modifications. Germ cells including spermatids were squeezed out from the seminiferous tubules onto slide glasses and incubated at 37°C until dry. The samples were fixed with 4% paraformaldehyde in PBS for 15 min. After three 5 min washes with PBS, the samples were permeabilized with 0.3% Triton X-100 for 15 min, washed with PBS three times for 5 min each, and blocked with 5% BSA and 10% goat serum diluted in PBS for 1 h at room temperature. Incubations with antibodies were performed as described above. Samples were observed using a Nikon Eclipse Ti microscope connected to a Nikon C2 confocal module (Nikon, Tokyo, Japan). Fluorescence images were false-colored and cropped using ImageJ software (version 2.0.0, NIH, Bethesda, MD, USA). Antibodies used are listed in S3 Table.

### Ultrastructural analysis using transmission electron microscope (TEM)

Testis samples were prepared as previously described [55]. The sections were observed using a JEM-1400 plus electron microscope (JEOL, Tokyo, Japan) at 80 kV with a CCD Veleta 2K × 2K camera (Olympus).

Brain and trachea samples were prepared as previously described [56] with slight modifications. Ultrathin sections stained with uranyl acetate solution for 5 min, briefly washed three times with distilled water, stained with Reynolds lead staining solution for 5 min, and washed three times with distilled water. The sections were observed using a JEM-1200EX electron microscope (JEOL) at 80 kV.

### Ultrastructural analysis using scanning electron microscope (SEM)

Brain and trachea samples were prepared as previously described [56]. The samples were observed using a JCM-5000 NeoScope Table Top SEM (JEOL).

### Ciliary motility analysis

Ependymal cilia were prepared as previously described [56]. The motility of ependymal cilia was observed under a 60x objective (LUCPlan FL N, Olympus) on a differential interference contrast microscope (BX53, Olympus) and recorded at 200 fps through a HAS-220 high-speed camera (DITECT, Tokyo, Japan).

### Intracytoplasmic sperm injection (ICSI)

Mature oocytes were collected from superovulated B6D2F1 female mice of 7–8 weeks of age, 13–15 h after injection of human chorionic gonadotropin (hCG, ASKA Animal Health, Tokyo, Japan). After treatment with 1 mg/ mL of hyaluronidase (088–07243, FUJIFILM Wako Pure Chemical) for 5 min to remove the cumulus cells, oocytes were placed in KSOM medium at 37°C under 5% CO_2_ in air until subjected to ICSI. Wild-type sperm heads were separated from the tail by applying a few piezo pulses. Because mutant spermatozoa proved difficult in separating the head, the whole spermatozoa was used for subsequent ICSI. The sperm head was injected into a mature oocyte using a piezo manipulator (PrimeTech, Tsuchiura, Ibaraki, Japan), as previously described [57]. The following day, two-cell embryos were transferred to pseudopregnant females. Genotyping was conducted one week after the birth.

### Statistical analysis

Statistical analyses were performed using a two-tailed student's *t*-test (*N* ≥ 3). Differences were considered significant at p<0.05 (*), p<0.01 (**), and p<0.001 (***). Data represent the means ± standard deviation (SD) and error bars indicate SD.

## Supporting information

S1 Fig Sequence similarity of FBXL13 in various organisms. Sequence similarity of FBXL13 in *Chlamydomonas reinhardtii*, *Trypanosoma brucei*, *Drosophila melanogaster*, *Xenopus tropicalis*, *Mus musculus*, and *Homo sapiens*. Dark purple indicates a match in all species. Blue indicates a match among four species. Light purple indicates a match among at least three species.
(TIF)

S2 Fig Sequence similarity of DRC7 in various organisms. Sequence similarity of the C-terminus of DRC7 in *Chlamydomonas reinhardtii*, *Trypanosoma brucei*, *Drosophila melanogaster*, *Xenopus tropicalis*, *Mus musculus*, and *Homo sapiens*. Dark purple indicates a match in all species. Blue indicates a match among four species. Light purple indicates a match among at least three species.
(TIF)

S3 Fig Generation of *Fbxl13* or *Drc7* knockout mice. (A) Targeting scheme for generating *Fbxl13* KO mice with the CRISPR/Cas9 system. Exon 1 and exon 7 were targeted. Magenta arrows indicate gRNA #1 and gRNA #2 that were used for targeting. The region flanked by these two gRNAs was deleted. Black arrowheads (Primer F1, F2, R1 and R2) indicate primers for genotyping, shown in (B). Genomic PCR with primers F2 and R2 can amplify the KO allele because of large deletion. Blue arrowheads (Primer F3 and R3) indicate primers for RT-PCR, shown in (C). (B) Genotyping of *Fbxl13* mutant mice. (C) mRNA expression of *Fbxl13* in *Fbxl13*^*+/-*^ and *Fbxl13*^*-/-*^ testis. No bands were detected in the *Fbxl13*^*-/-*^ testis. (D) Targeting scheme for generating *Drc7* KO mice using a vector obtained from the Knockout Mouse Project (KOMP). LacZ-neo expression cassette was introduced into intron 8. Mice with a tm1a allele were mated with CAG-Cre transgenic mice to delete the region flanked by loxP sites including neo expression cassette and exon 9–11. Black arrowheads (Primer F1, F2, R1, R2 and R3) indicate primers for genotyping and RT-PCR, shown in (E) and (F). En2 SA, Engrailed-2 splice acceptor; IRES, internal ribosome entry site; pA, SV40 polyadenylation signal; hBactP, human β-actin promoter (an autonomous promoter); neo, neomycin resistance gene; DTA, Diphtheria Toxin A. (E) Genotyping of *Drc7* mutant mice. (F) mRNA expression of *Drc7* in wild-type and *Drc7* KO testis. No bands were detected in the *Drc7*^*-/-*^ testis.
(TIF)

S4 Fig *Fbxl13* is not required for male fertility. (A) Number of pups born per plug. *Fbxl13*^*-/-*^ male mice sired offspring comparable to wild-type males. (B) PAS staining of seminiferous tubules of adult mice. Testis morphology of *Fbxl13*^*-/-*^ mice is comparable to that of *Fbxl13*^*+/-*^ mice. Scale bar, 100 μm. (C) Observation of spermatozoa obtained from the cauda epididymis. *Fbxl13* KO spermatozoa exhibit normal head and tail morphology. Scale bar, 50 μm. (D) Sperm motility analyzed with Computer-Assisted Sperm Analysis (CASA). Error bars represent S.D. No significant differences were found in all the parameters (unpaired Student's t-test). *N* = 5 males each for *Fbxl13*^*+/-*^ and *Fbxl13*^*-/-*^ mice.
(TIF)

S5 Fig *Fbxl13* and *Drc7* are not required for ciliogenesis in the trachea. (A) Observation of wild-type, *Fbxl13*^*-/-*^, and *Drc7*^*-/-*^ tracheal cilia using scanning electron microscopy. Morphology of *Fbxl13*^*-/-*^ or *Drc7*^*-/-*^ cilia is comparable to that of wild-type cilia. Scale bar, 5 μm. (B) Observation of wild-type, *Fbxl13*^*-/-*^, and *Drc7*^*-/-*^ tracheal cilia using transmission electron microscopy. The ‘9+2’ structure with both inner and outer dynein arms was found in *Fbxl13*^*-/-*^ and *Drc7*^*-/-*^ mice. Scale bar, 100 nm.
(TIF)

S6 Fig *Drc7* is not required for ciliogenesis in the brain. (A) Observation of wild-type and *Drc7*^*-/-*^ ependymal cilia using scanning electron microscopy. Morphology of *Drc7*^*-/-*^ cilia is comparable to that of wild-type cilia. Scale bar, 5 μm. (B) Observation of wild-type and *Drc7*^*-/-*^ ependymal cilia using transmission electron microscopy. The ‘9+2’ structure with both inner and outer dynein arms was found in both wild-type and *Drc7*^*-/-*^ mice. Scale bar, 100 nm.
(TIF)

S1 Table N-DRC information. (TIF)

S2 Table Sequences of primers. (TIF)

S3 Table Antibodies used in this study. (TIF)

S1 Movie Sperm motility of *Drc7*^*+/-*^ mice. Scale bar, 20 μm.
(AVI)

S2 Movie Sperm motility of *Drc7*^*-/-*^ mice. Scale bar, 20 μm.
(AVI)

S3 Movie Ependymal cilia motility in wild-type mice. Scale bar, 10 μm.
(AVI)

S4 Movie Ependymal cilia motility in *Drc7*^*-/-*^ mice. Scale bar, 10 μm.
(AVI)
