# Supplementary figures and images for: Nexin-Dynein regulatory complex component DRC7 but not FBXL13 is required for sperm flagellum formation and male fertility in mice
Source: PLoS Genet. 2020 Jan 21;16(1):e1008585. doi: 10.1371/journal.pgen.1008585 (PMC6994161; doi:10.1371/journal.pgen.1008585)

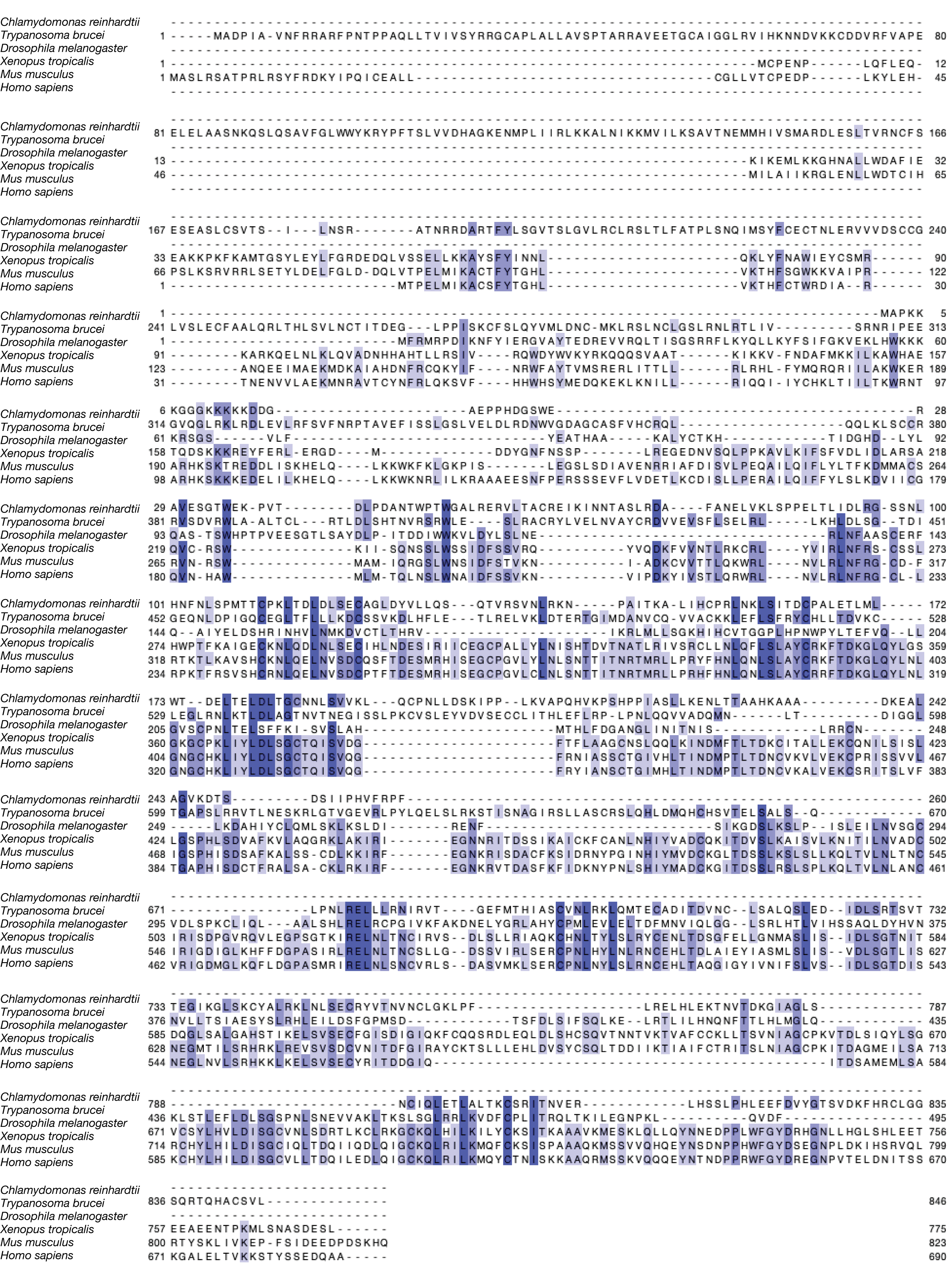

Supplement: S1 Fig — Sequence similarity of FBXL13 in Chlamydomonas reinhardtii, Trypanosoma brucei, Drosophila melanogaster, Xenopus tropicalis, Mus musculus, and Homo sapiens. Dark purple indicates a match in all species. Blue indicates a match among four species. Light purple indicates a match among at least three species. (TIF) [file pgen.1008585.s001.tif]

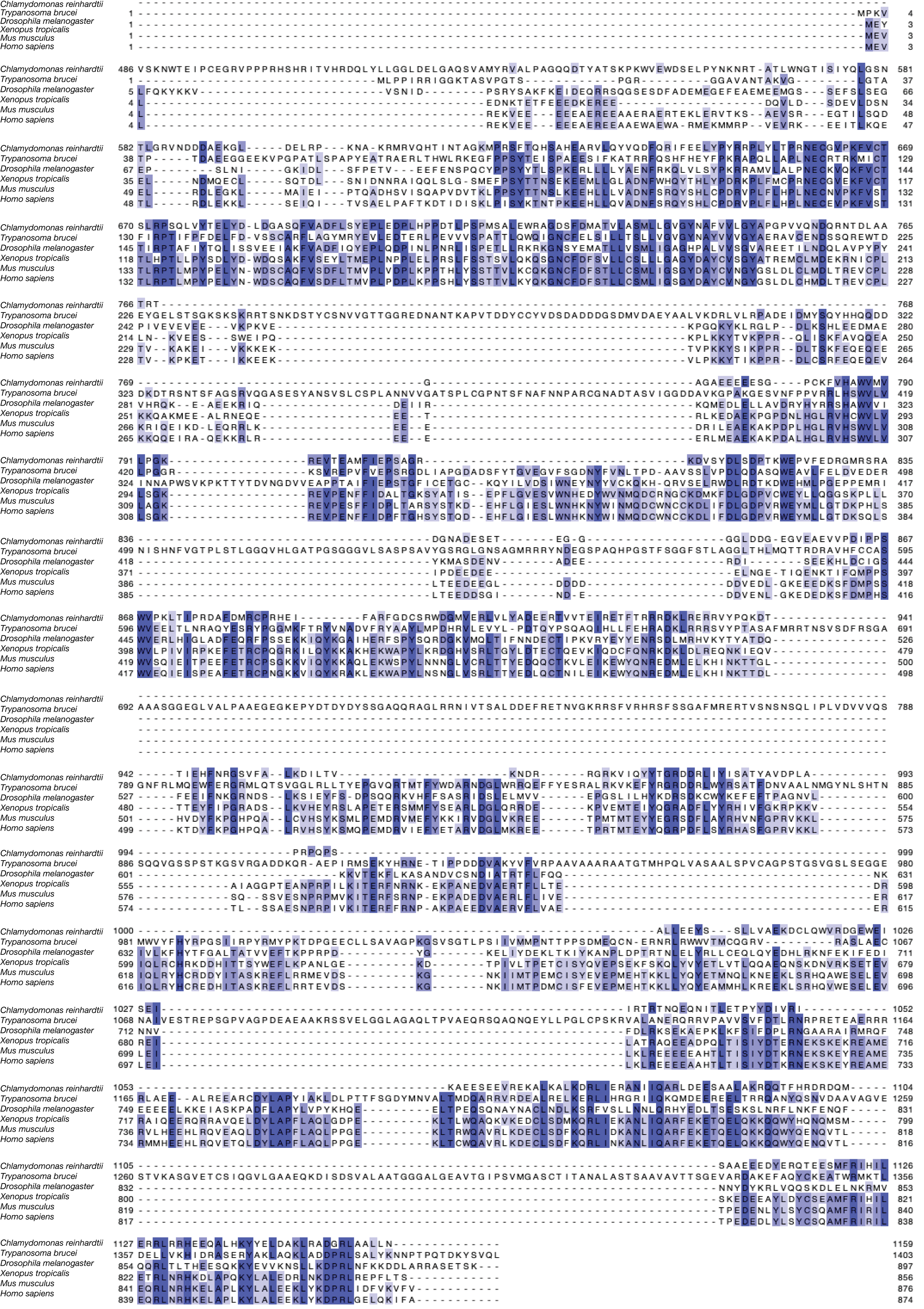

Supplement: S2 Fig — Sequence similarity of the C-terminus of DRC7 in Chlamydomonas reinhardtii, Trypanosoma brucei, Drosophila melanogaster, Xenopus tropicalis, Mus musculus, and Homo sapiens. Dark purple indicates a match in all species. Blue indicates a match among four species. Light purple indicates a match among at least three species. (TIF) [file pgen.1008585.s002.tif]

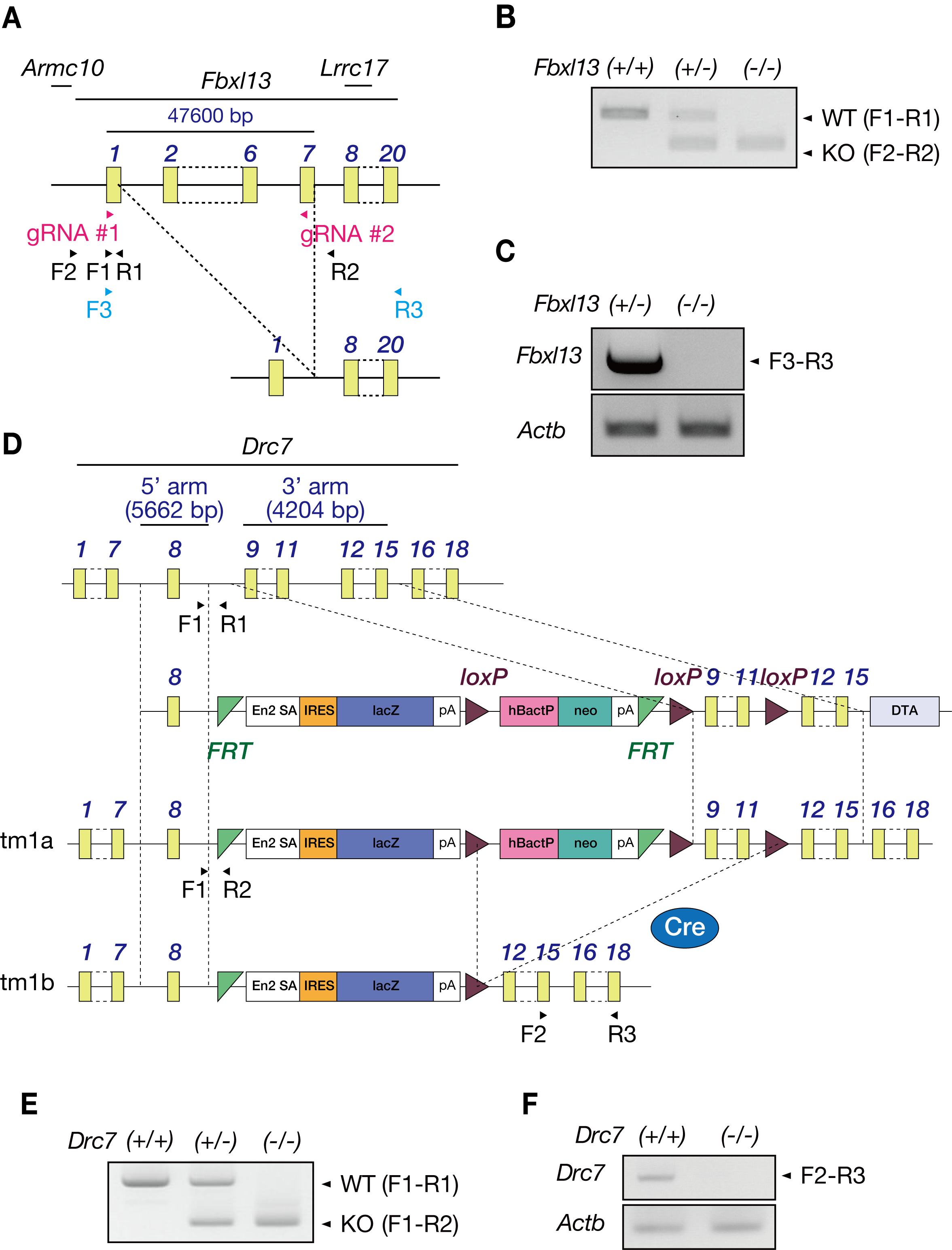

Supplement: S3 Fig — (A) Targeting scheme for generating Fbxl13 KO mice with the CRISPR/Cas9 system. Exon 1 and exon 7 were targeted. Magenta arrows indicate gRNA #1 and gRNA #2 that were used for targeting. The region flanked by these two gRNAs was deleted. Black arrowheads (Primer F1, F2, R1 and R2) indicate primers for genotyping, shown in (B). Genomic PCR with primers F2 and R2 can amplify the KO allele because of large deletion. Blue arrowheads (Primer F3 and R3) indicate primers for RT-PCR, shown in (C). (B) Genotyping of Fbxl13 mutant mice. (C) mRNA expression of Fbxl13 in Fbxl13+/- and Fbxl13-/- testis. No bands were detected in the Fbxl13-/- testis. (D) Targeting scheme for generating Drc7 KO mice using a vector obtained from the Knockout Mouse Project (KOMP). LacZ-neo expression cassette was introduced into intron 8. Mice with a tm1a allele were mated with CAG-Cre transgenic mice to delete the region flanked by loxP sites including neo expression cassette and exon 9–11. Black arrowheads (Primer F1, F2, R1, R2 and R3) indicate primers for genotyping and RT-PCR, shown in (E) and (F). En2 SA, Engrailed-2 splice acceptor; IRES, internal ribosome entry site; pA, SV40 polyadenylation signal; hBactP, human β-actin promoter (an autonomous promoter); neo, neomycin resistance gene; DTA, Diphtheria Toxin A. (E) Genotyping of Drc7 mutant mice. (F) mRNA expression of Drc7 in wild-type and Drc7 KO testis. No bands were detected in the Drc7-/- testis. (TIF) [file pgen.1008585.s003.tif]

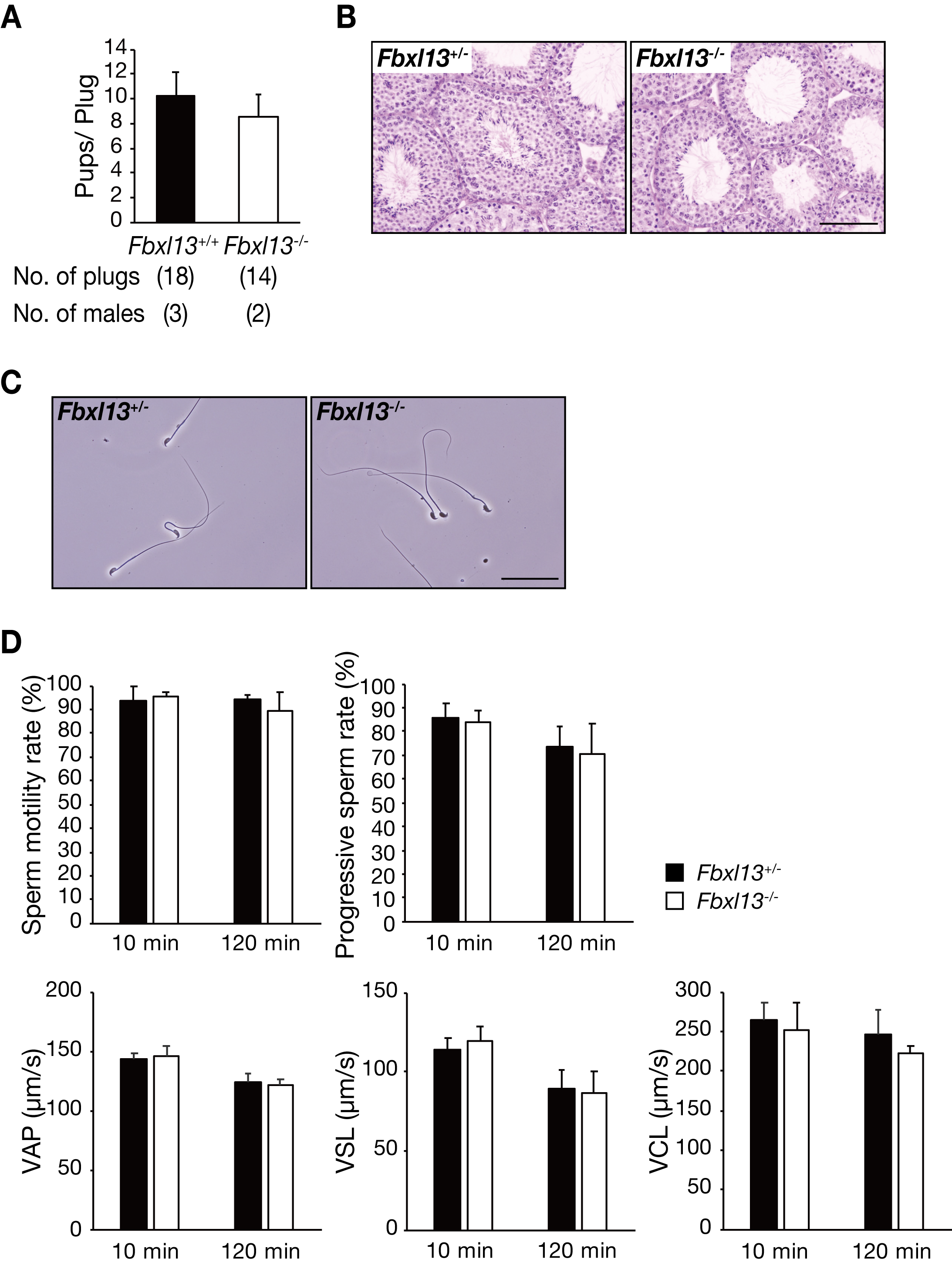

Supplement: S4 Fig — (A) Number of pups born per plug. Fbxl13-/- male mice sired offspring comparable to wild-type males. (B) PAS staining of seminiferous tubules of adult mice. Testis morphology of Fbxl13-/- mice is comparable to that of Fbxl13+/- mice. Scale bar, 100 μm. (C) Observation of spermatozoa obtained from the cauda epididymis. Fbxl13 KO spermatozoa exhibit normal head and tail morphology. Scale bar, 50 μm. (D) Sperm motility analyzed with Computer-Assisted Sperm Analysis (CASA). Error bars represent S.D. No significant differences were found in all the parameters (unpaired Student's t-test). N = 5 males each for Fbxl13+/- and Fbxl13-/- mice. (TIF) [file pgen.1008585.s004.tif]

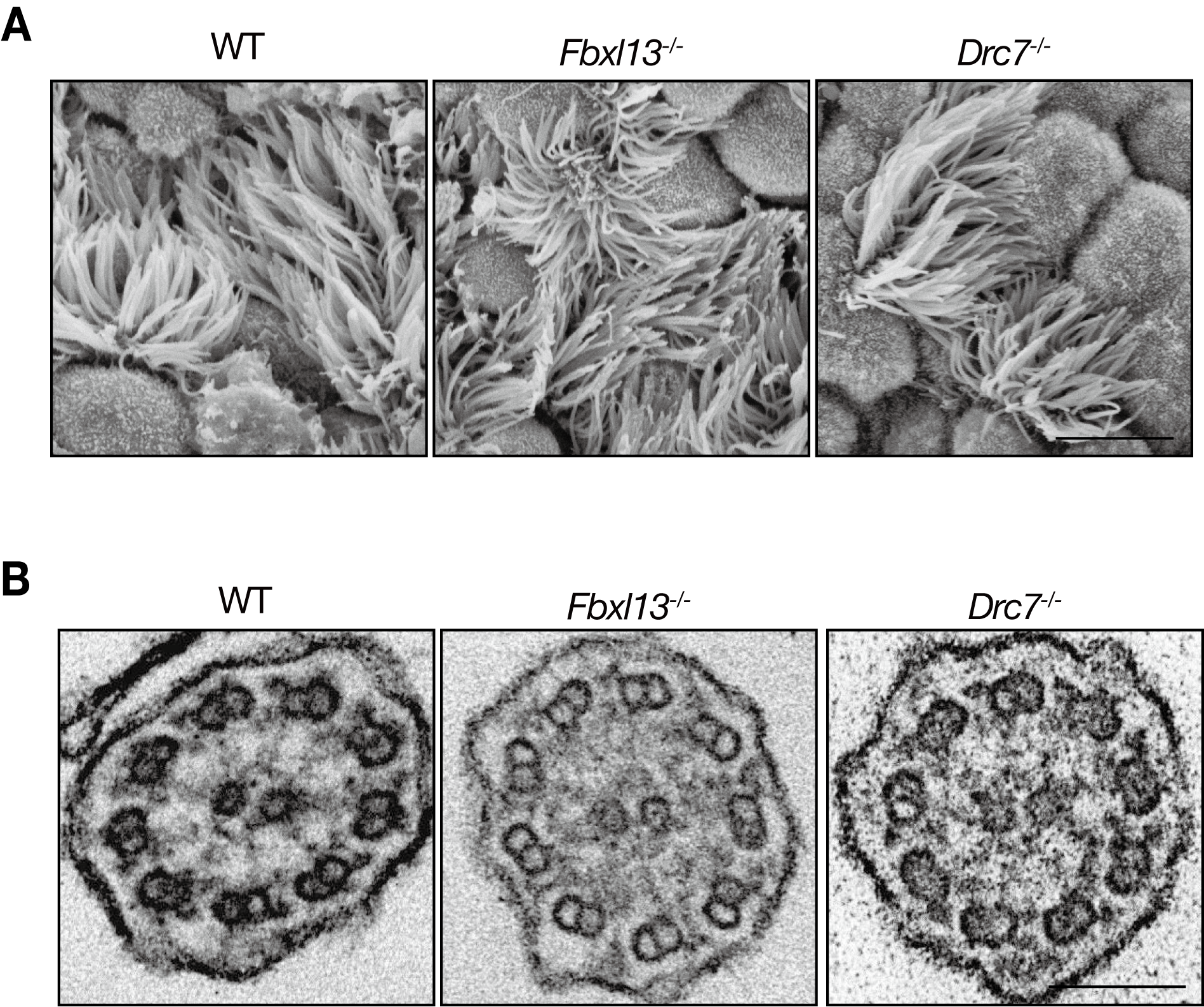

Supplement: S5 Fig — (A) Observation of wild-type, Fbxl13-/-, and Drc7-/- tracheal cilia using scanning electron microscopy. Morphology of Fbxl13-/- or Drc7-/- cilia is comparable to that of wild-type cilia. Scale bar, 5 μm. (B) Observation of wild-type, Fbxl13-/-, and Drc7-/- tracheal cilia using transmission electron microscopy. The ‘9+2’ structure with both inner and outer dynein arms was found in Fbxl13-/- and Drc7-/- mice. Scale bar, 100 nm. (TIF) [file pgen.1008585.s005.tif]

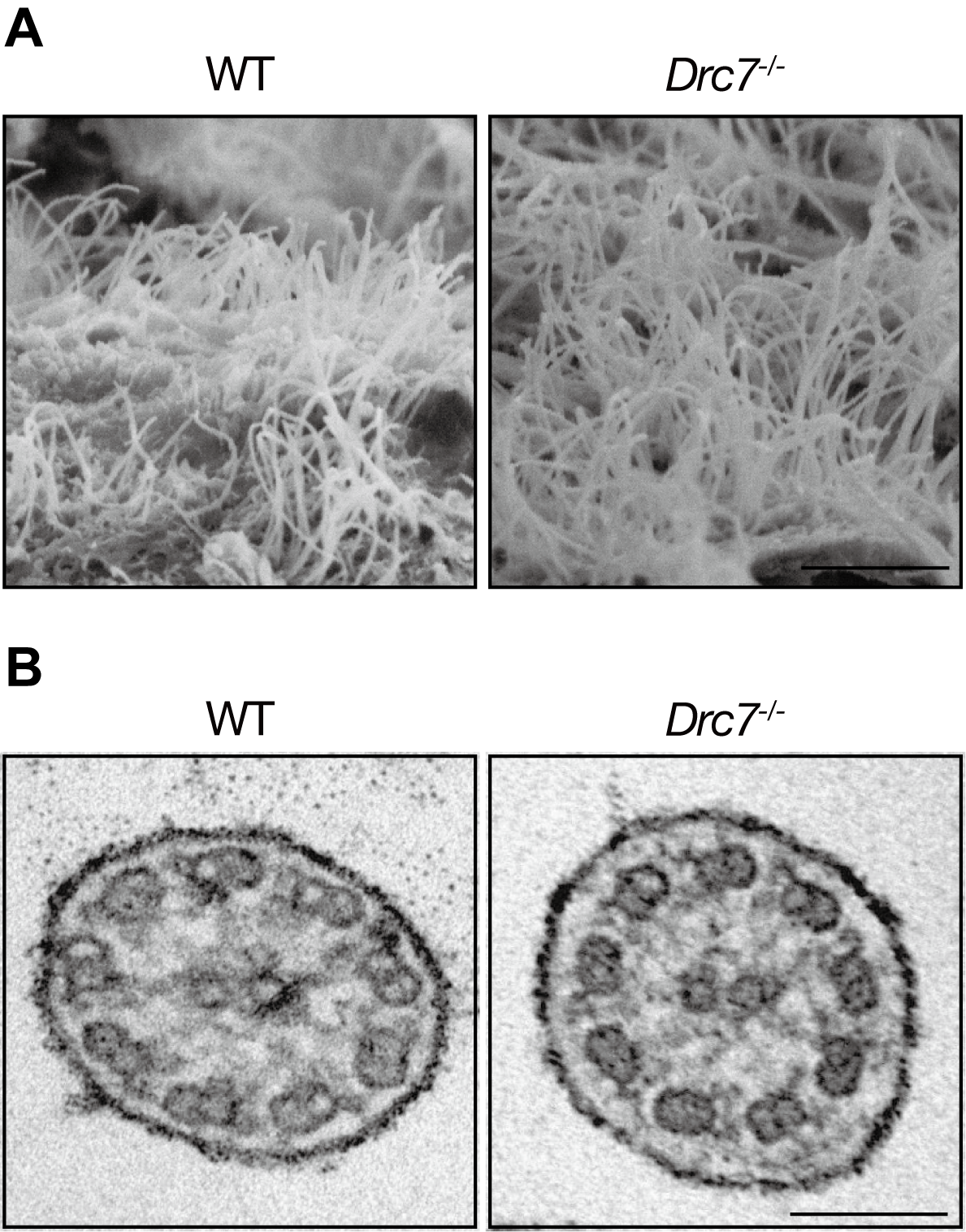

Supplement: S6 Fig — (A) Observation of wild-type and Drc7-/- ependymal cilia using scanning electron microscopy. Morphology of Drc7-/- cilia is comparable to that of wild-type cilia. Scale bar, 5 μm. (B) Observation of wild-type and Drc7-/- ependymal cilia using transmission electron microscopy. The ‘9+2’ structure with both inner and outer dynein arms was found in both wild-type and Drc7-/- mice. Scale bar, 100 nm. (TIF) [file pgen.1008585.s006.tif]

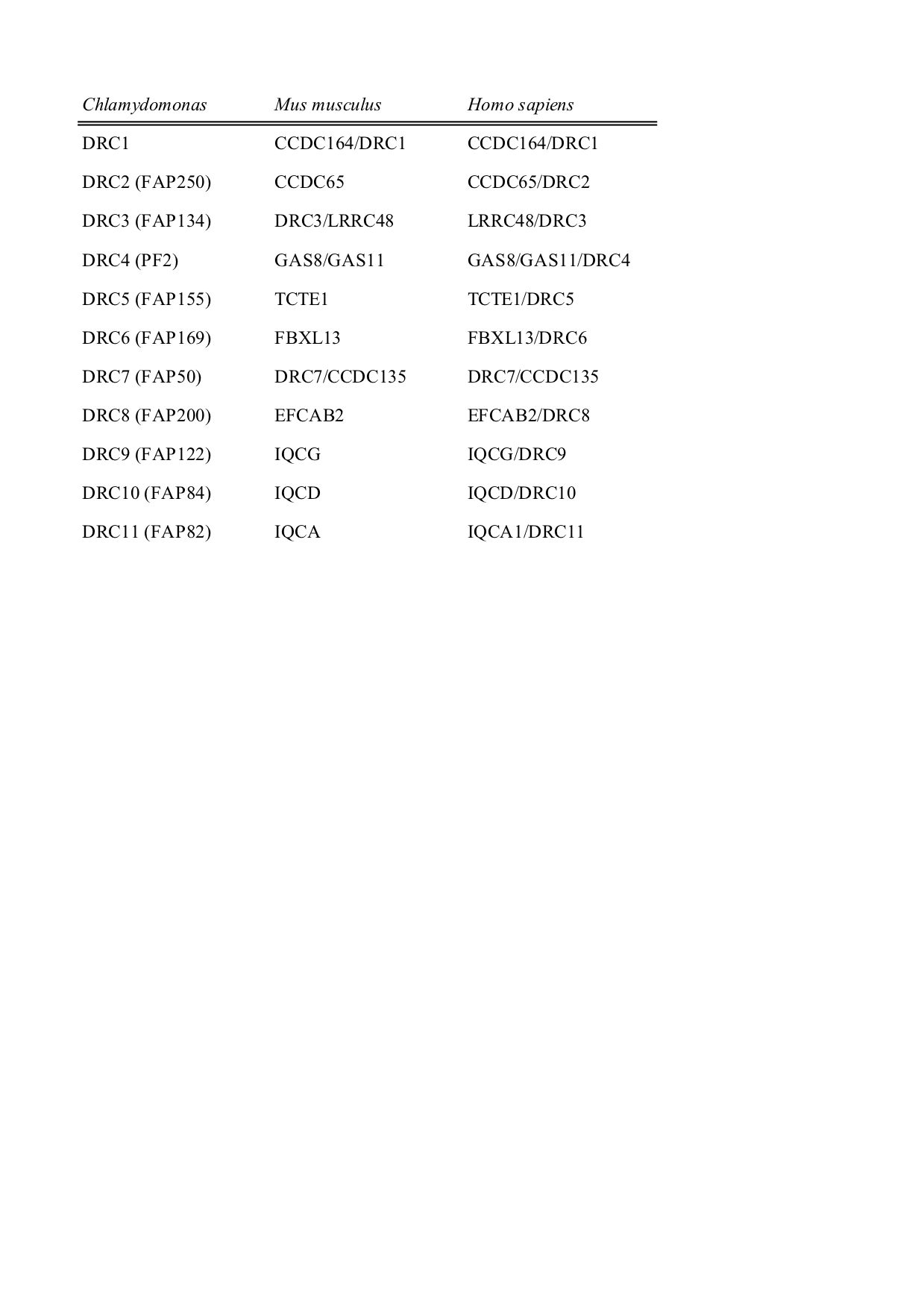

Supplement: S1 Table — (TIF) [file pgen.1008585.s007.tif]

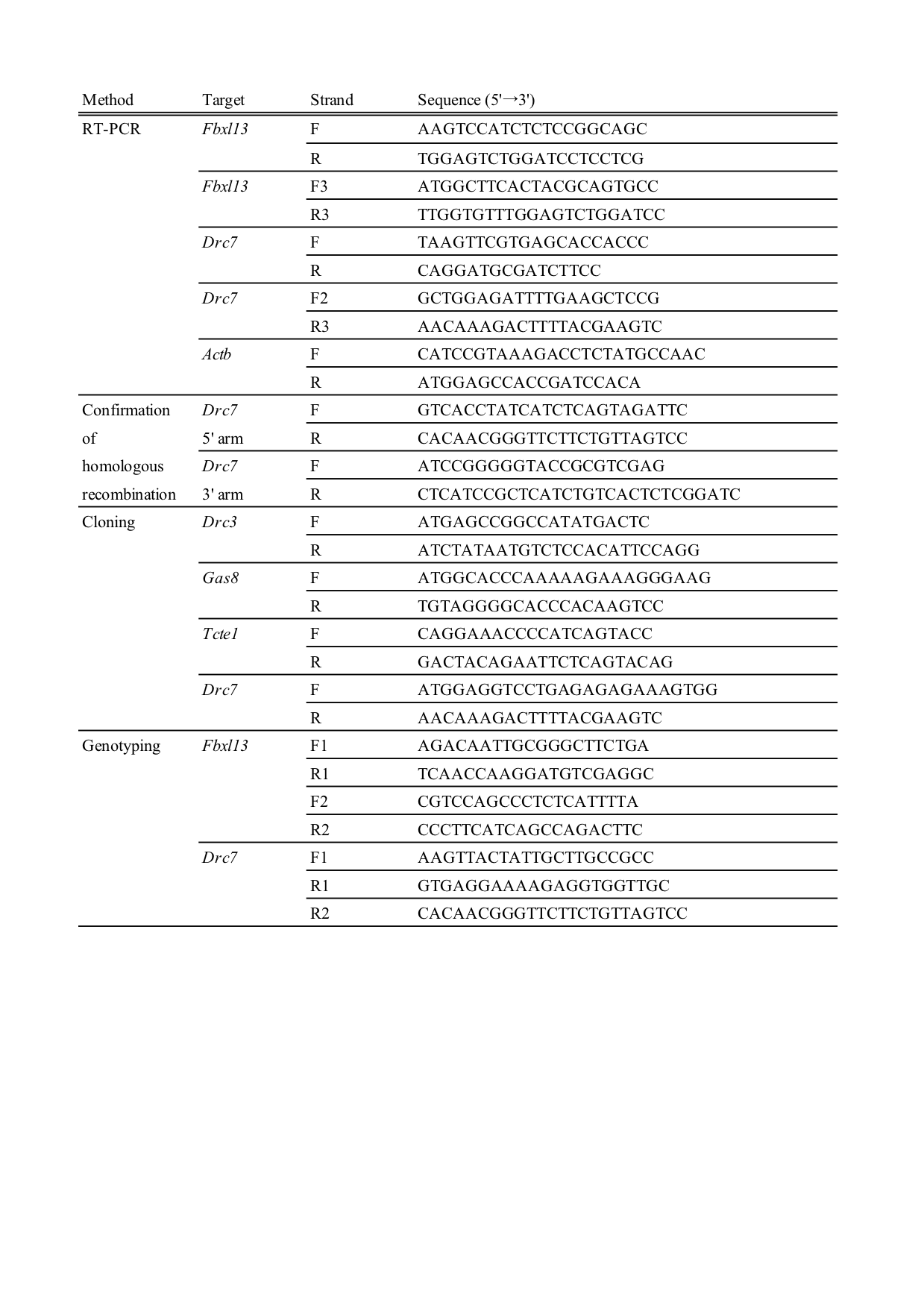

Supplement: S2 Table — (TIF) [file pgen.1008585.s008.tif]

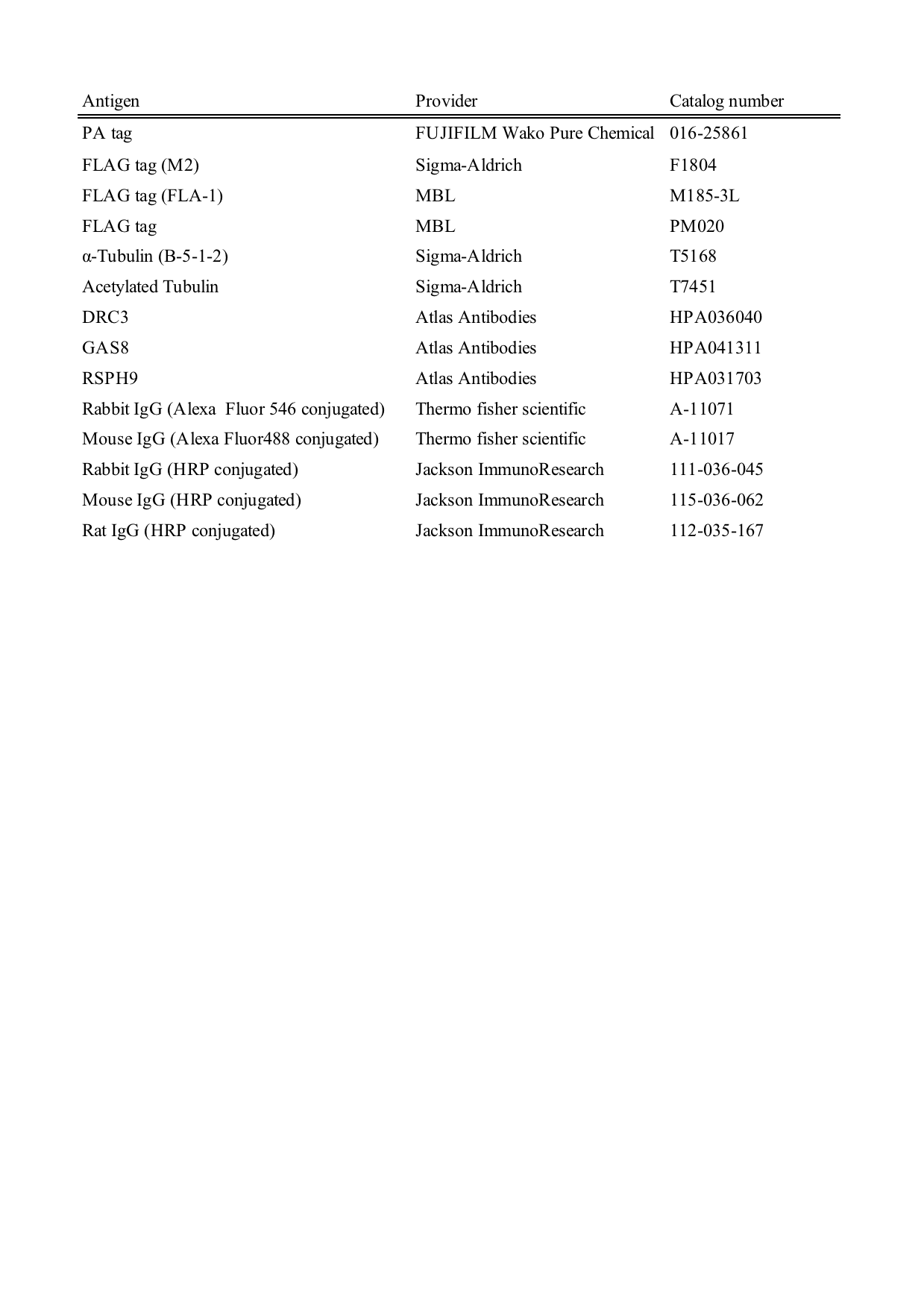

Supplement: S3 Table — (TIF) [file pgen.1008585.s009.tif]
